# Abnormal Cholesterol Metabolism and Lysosomal Dysfunction Induce Age-Related Hearing Loss by Inhibiting mTORC1-TFEB-Dependent Autophagy

**DOI:** 10.3390/ijms242417513

**Published:** 2023-12-15

**Authors:** Yun Yeong Lee, Jungho Ha, Young Sun Kim, Sivasubramanian Ramani, Siung Sung, Eun Sol Gil, Oak-Sung Choo, Jeong Hun Jang, Yun-Hoon Choung

**Affiliations:** 1 Department of Otolaryngology, Ajou University School of Medicine, Suwon 16499, Republic of Korea; seven260@naver.com (Y.Y.L.); jhflyflyfly@ajou.ac.kr (J.H.); jmyea@hanmail.net (Y.S.K.); drshivaha@outlook.com (S.R.); ylem2s1u@gmail.com (S.S.); jhj@ajou.ac.kr (J.H.J.); 2 Department of Medical Sciences, Ajou University Graduate School of Medicine, Suwon 16499, Republic of Korea; 3 Department of Otorhinolaryngology-Head and Neck Surgery, Kangnam Sacred Heart Hospital, Hallym University College of Medicine, Seoul 07441, Republic of Korea; oschoo1202@gmail.com

**Keywords:** age-related hearing loss, autophagy, hearing loss, lysosome biogenesis, mTORC1, transcription factor

## Abstract

Cholesterol is a risk factor for age-related hearing loss (ARHL). However, the effect of cholesterol on the organ of Corti during the onset of ARHL is unclear. We established a mouse model for the ARHL group (24 months, *n* = 12) and a young group (6 months, *n* = 12). Auditory thresholds were measured in both groups using auditory brainstem response (ABR) at frequencies of 8, 16, and 32 kHz. Subsequently, mice were sacrificed and subjected to histological analyses, including transmission electron microscopy (TEM), H&E, Sudan Black B (SBB), and Filipin staining, as well as biochemical assays such as IHC, enzymatic analysis, and immunoblotting. Additionally, mRNA extracted from both young and aged cochlea underwent RNA sequencing. To identify the mechanism, in vitro studies utilizing HEI-OC1 cells were also performed. RNA sequencing showed a positive correlation with increased expression of genes related to metabolic diseases, cholesterol homeostasis, and target of rapamycin complex 1 (mTORC1) signaling in the ARHL group as compared to the younger group. In addition, ARHL tissues exhibited increased cholesterol and lipofuscin aggregates in the organ of Corti, lateral walls, and spiral ganglion neurons. Autophagic flux was inhibited by the accumulation of damaged lysosomes and autolysosomes. Subsequently, we observed a decrease in the level of transcription factor EB (TFEB) protein, which regulates lysosomal biosynthesis and autophagy, together with increased mTORC1 activity in ARHL tissues. These changes in TFEB and mTORC1 expression were observed in a cholesterol-dependent manner. Treatment of ARHL mice with atorvastatin, a cholesterol synthesis inhibitor, delayed hearing loss by reducing the cholesterol level and maintaining lysosomal function and autophagy by inhibiting mTORC1 and activating TFEB. The above findings were confirmed using stress-induced premature senescent House Ear Institute organ of Corti 1 (HEI-OC1) cells. The findings implicate cholesterol in the pathogenesis of ARHL. We propose that atorvastatin could prevent ARHL by maintaining lysosomal function and autophagy by inhibiting mTORC1 and activating TFEB during the aging process.

## 1. Introduction

Cholesterol is important for cell membrane structure and function and for hormone and vitamin synthesis in mammals. However, deregulation of cholesterol homeostasis is a characteristic of chronic neurodegenerative diseases such as Parkinson’s disease, Alzheimer’s disease, and Huntington’s disease [1].

There is a relationship between cholesterol homeostasis deregulation and sensorineural hearing loss (SNHL). Niemann–Pick syndrome type C (NPC) affects cholesterol intracellular transport, thereby disturbing neurological phenotypes including SNHL [2]. Hyper-cholesterolemia and -lipidemia predispose to SNHL [3,4,5,6], while atherosclerosis and a low high-density lipid (HDL) level are also positively correlated with SNHL. Low-density lipid receptor (LDLR) shows expression in cochlear cells associated with endolymph homeostasis, and following the onset of hearing, it is also expressed in non-sensory supporting cells, potentially establishing a mechanical coupling with adjacent outer hair cells to modulate electromotility and cochlear amplification [7]. Therefore, therapies that reduce the plasma cholesterol (or lipid) level could prevent SNHL. For instance, some statins exert an otoprotective effect by improving cochlear microcirculation and oxidative stress [5,8,9,10]. Hypercholesterolemia is a risk factor for hearing loss, but the underlying molecular mechanisms are unclear.

The master regulator of the autophagy-lysosomal pathway is transcription factor EB (TFEB), a member of the MiT family of transcription factors, together with TFE3, TFEC, and MITF [11]. Nuclear translocation of TFEB induces lysosomal biogenesis and upregulation of the autophagic core process machinery. Moreover, mitochondrial turnover is dependent on the autophagy-lysosomal pathway. Damaged mitochondria produce a signal that triggers nuclear translocation of TFEB [12,13,14,15].

The master growth regulator, mammalian target of rapamycin complex 1 (mTORC1) kinase, is activated in the lysosome. The lysosome is also a major sorting station for dietary cholesterol. Lysosomal cholesterol drives mTORC1 activation and growth signaling via the SLC38A9-NPC1 complex. Moreover, mTORC1 redistributes TFEB from the nucleus to the cytoplasm upon resumption of low-density lipid (LDL) supply after sterol depletion in HEK-293T and CHO cells stably expressing TFEB-GFP protein [16].

The activation of mTORC1 phosphorylates TFEB at serine S211 to promote its dissociation from 14-3-3 protein family members, which retain TFEB in the cytoplasm and inhibit its transcriptional activity. Moreover, mTORC1 negatively regulates TFEB protein levels [17,18,19,20,21].

Autophagic dysfunction-mediated sensory epithelial cell loss and spiral ganglion neuron (SGN) degeneration are induced by inhibition of nuclear migration of TFEB in a model of hearing loss caused by the ototoxic drugs, kanamycin and furosemide. Therefore, TFEB may be a target for attenuating SGN degeneration following sensory epithelial cell loss in ototoxic drug-induced hearing loss [22].

ARHL is one of the most common disorders affecting older adults. The factors contributing to ARHL are very diverse, such as noise, ototoxic drugs, mitochondrial DNA mutation, and genetic disorders. Especially, cardiovascular risk factors (e.g., hypertension, diabetes, smoking, or increased serum cholesterol) exhibit a high risk of the onset of ARHL [23,24,25]. However, the mechanisms underlying these processes remain unclear in ARHL.

The purposes of the study were to investigate the association of pathogenic mechanisms between metabolic syndrome (hypercholesterolemia) and ARHL, as well as to analyze the mechanism of action of atorvastatin as a prevention medication against ARHL.

## 2. Results

### 2.1. Metabolic Disease-Related Gene Expression in Cochlear Tissues

**Figure 1:**
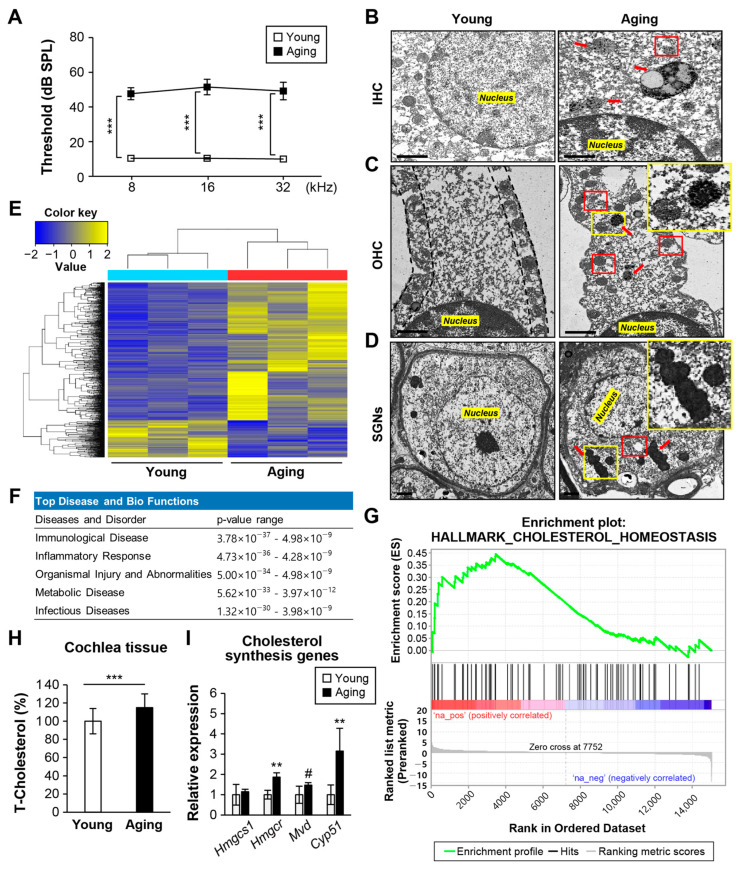
Cochlea of mice with ARHL show increased cholesterol level, cholesterol synthesis, and lipofuscin aggregation. (**A**) The ABR threshold at 8, 16, and 32 kHz was significantly higher in the ARHL group (24 months, *n* = 12) than in the young group (6 months, *n* = 12). Means ± SEM. TEMs of (**B**) inner hair cells, (**C**) outer hair cells, and (**D**) SGNs in the ARHL and young cochleae. Red boxes, autolysosome accumulation. Abnormal enlarged lysosome, lipofuscin aggregates (red arrows or yellow box, higher-magnification images) in the ARHL and young groups (*n* = 3 for each group). Scale bar, 1 μm. (**E**) Heat map of 931 DEGs in the young and ARHL groups. (**F**) Top diseases and disorders by IPA of DEGs between young and ARHL groups. (**G**) GSEA results show DEGs in the ARHL group as compared to the young group. Ranked list was generated using the FC and raw *p*-values between the ARHL and young groups. (**H**) Total cholesterol levels in cochlear extracts (*n* = 3 for each group). (**I**) qPCR analysis of cholesterol synthesis-related genes. *18S ribosomal RNA* was used as the control. *n* = 3 for each group. Means ± SD. The heat map, IPA, and GSEA source data are provided as a Supplementary source file. Experiments were performed in at least duplicate # *p* < 0.05, ** *p* < 0.01, *** *p* < 0.001 (Student’s *t*-test or one-way ANOVA, followed by Tukey’s HSD test).

The ABR thresholds at 8, 16, and 32 kHz were significantly increased by more than 40 dB in the aged group (24 M) as compared to the young (6 M) group, confirming the onset of ARHL (Figure 1A). Electron microscopy revealed that the organ of Corti in the ARHL group exhibited abnormal lysosome, lipofuscin, and autolysosome accumulation in inner hair cells (IHCs), outer hair cells (OHCs), and SGNs (Figure 1B–D). This is similar to the report that SGN degeneration and sensory epithelial cell loss are caused by autophagic dysfunction and accumulation of lipofuscin in mice with kanamycin and furosemide-induced hearing loss [22]. To investigate the cause of lipofuscin accumulation in ARHL tissues, RNA sequencing (RNA-seq) analysis was performed on cochlear extracts from young and ARHL mice. The ARHL group showed a marked change in gene expression as compared to the young group (Figure 1E). Ingenuity pathway analysis (IPA) showed that the expression levels of genes related to immunological disease, inflammatory response, organismal injury, and abnormalities were higher in the ARHL group as compared to the young group. Also, high expression of metabolic disease-related genes was observed (Figure 1F). The changes in the expression of genes enhancing cholesterol homeostasis were positively correlated with the gene set enrichment assay (GSEA) results (Figure 1G). This was consistent with the increased total cholesterol level and expression of cholesterol synthesis-related genes in cochlear tissues (Figure 1H,I). Similarly, the expression of genes that increase fatty acid metabolism and lipid synthesis-related genes was higher in the ARHL group than in the young group (Supplementary Figure S1A,B).

Consequently, changes in cochlear tissue metabolism in the ARHL group led to abnormal cholesterol and lipid overproduction, which were not degraded by the autophagy-lysosomal system.

### 2.2. mTORC1 Activity, TFEB Expression, Lysosomal Damage, and Autophagic Flux Inhibition in Cochlear Tissues

**Figure 2:**
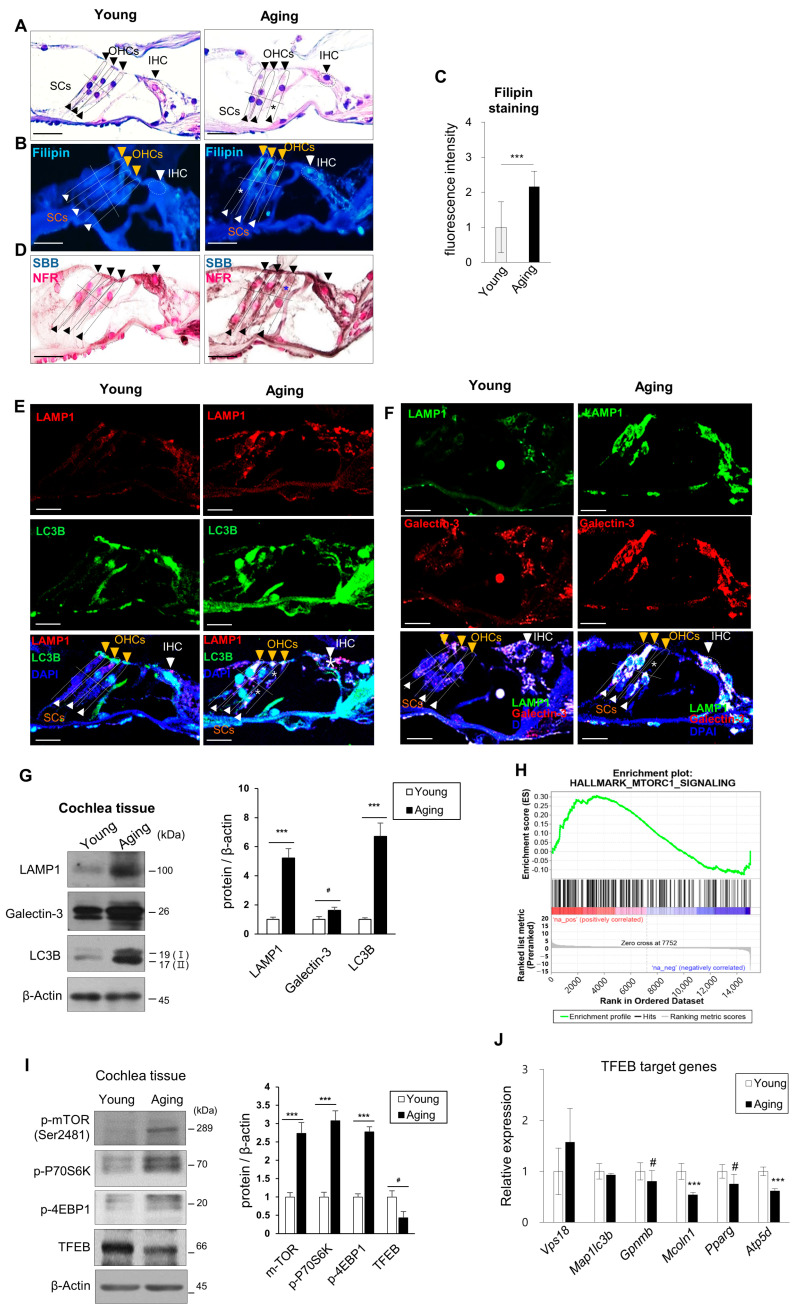
ARHL shows inhibited autophagic flux with lysosomal dysfunction, mTORC1 activation, and TFEB downregulation. (**A**) H&E-stained sections from the middle turns of the cochlea. Scale bars, 20 μm. (**B**) Filipin or (**D**) SBB staining, with NFR counterstaining, of cochlear sections from young and ARHL mice. Scale bars, 20 μm. (**C**) Filipin staining intensity quantification using ImageJ software (pixel intensity, *n* = 3 replicates from three independent cochlea samples). Sections were immunolabeled with anti-LAMP1, -LC3B, -LAMP1 and -galectin-3 antibodies to evaluate inhibition of autophagic flux by (**E**) autolysosome accumulation and (**F**) lysosomal dysfunction. DAPI was used as a counterstain. *n* = 3 for each group. Scale bars, 20 μm. (**G**) LAMP1, LC3B, and galectin-3 levels in whole cochlear fractions analyzed by immunoblotting. β-Actin was used as the loading control. (**H**) GSEA shows that the increased expression of mTORC1-related genes was detected in the ARHL group compared to the young group. (**I**) mTORC1 activity as measured by phosphorylation of mTORC1 and its substrates P70S6K and 4EBP1 as well as TFEB expression in whole cochlear fractions. β-Actin was used as the loading control. *n* = 3 for each group. (**J**) qPCR analysis of TFEB target genes. *18S ribosomal RNA* was used as the control. Black (also white, yellow) arrowheads indicate IHCs, OHCs, and supporting cells (SCs). Asterisks indicate cell loss. Means ± SD. *n* = 3 for each group. Experiments were performed at least three times for each condition and repeated at least twice. # *p* < 0.05, *** *p* < 0.001 (Student’s *t*-test or one-way ANOVA, followed by Tukey’s HSD test).

Loss of IHC, OHCs, and SGNs, decreased lateral cell wall density, stria vascularis atrophy, and blood vessel abnormalities were observed in the ARHL group. This was consistent with prior studies on the morphology of ARHL (Figure 2A and Supplementary Figure S1C–E) [26].

The Filipin cholesterol staining showed a significant increase in the ARHL group compared to the young group, with an elevation in fluorescence brightness indicating the accumulation of cholesterol in hair cells (Figure 2B,C and Supplementary Figure S2A,B). Lipofuscin accumulation (Figure 2D), autophagic flux inhibition (LC3B/LAMP1, Figure 2E), and damaged lysosome accumulation (Galectin-3/LAMP1, Figure 2F) were noted in ARHL tissues upon SBB staining and immunohistochemical analysis. Protein levels were confirmed by immunoblotting (Figure 2G). Accumulation of LC3B, LAMP1, and galectin-3 proteins was predictive of autophagic flux inhibition and impaired lysosome accumulation.

Accumulation of cholesterol and glycosphingolipids in late endosomes and lysosomes occurs in mutant NPC1 cells [27]. qPCR showed that *Npc1* expression was significantly decreased and *Npc2* expression was increased in the ARHL group (Supplementary Figure S3A,C). This is likely to be responsible for the lysosomal accumulation of cholesterol.

Accumulated cholesterol in lysosomes induces mTORC1 hyperactivation in the lysosomal membrane and inhibits autophagy/mitophagy and lysosomal and mitochondrial biogenesis [27]. The GSEA results showed that the increase in mTORC1 signaling-related gene groups was correlated with the inhibition of autophagy (LC3B, LAMP1) and increased damaged lysosome (LAMP1, galectin-3) (Figure 2H).

The phosphorylated mTORC1, P70S6K, and 4EBP1 levels were increased in ARHL cochlear tissues. Also, the expression of TFEB transcription targets decreased as the TFEB protein level decreased (Figure 2I,J and Supplementary Figure S3D,E).

Therefore, lipid and cholesterol levels, which were abnormally increased in the cochlear tissues of the ARHL group, accumulated due to impaired lysosomal function, causing mTORC1 hyperactivation. Lysosome- and autophagy-related protein turnover was suppressed by reducing the TFEB level and activity.

### 2.3. In Vivo Phenotype and Stress-Induced Premature Senescence Models of HEI-OC1 Cells

**Figure 3:**
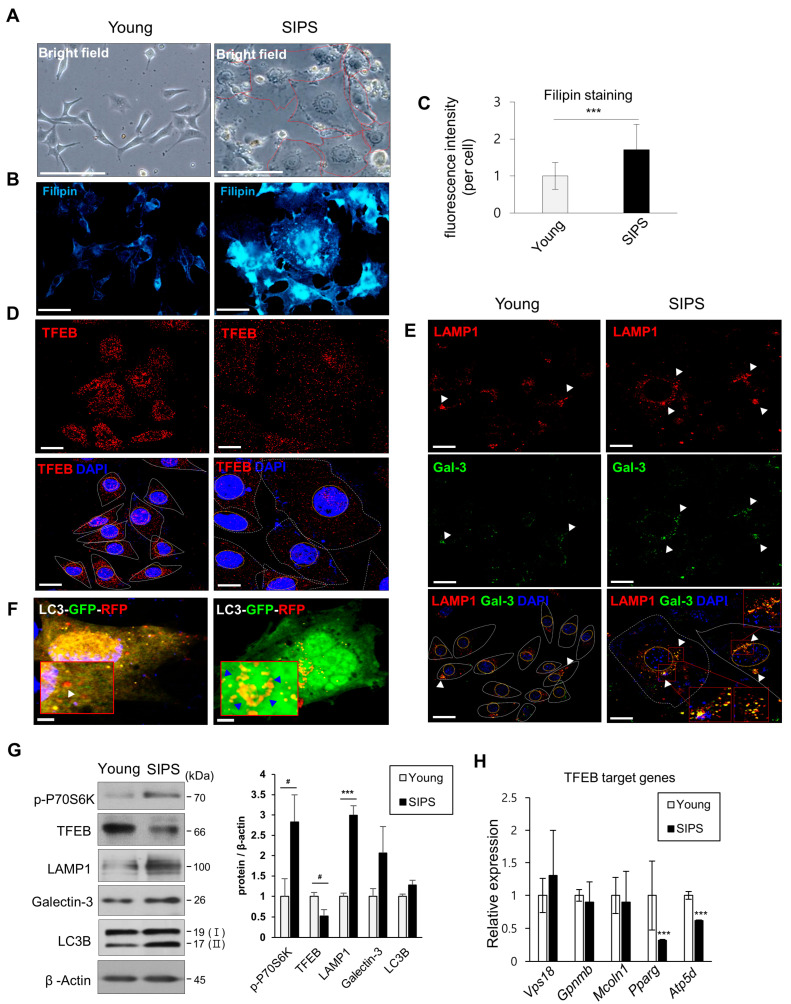
Decreases in TFEB expression were accompanied by increased cholesterol and mTORC1 activity markers and autophagic flux inhibition in SIPS. (**A**) HEI-OC1 cells were treated with DOXO (100 ng/mL) and maintained for 5 days. SIPS cells were enlarged. Scale bars, 100 μm. (**B**) Filipin staining of the cholesterol content of young and SIPS cells. Scale bars, 50 μm (**C**) Fluorescence micrographs were captured and at least 20 cells per image (5 images per group) were analyzed using ImageJ software. (**D**,**E**) TFEB expression and localization in young and SIPS cells and lysosomal dysfunction (LAMP1, galectin-3 (arrowheads)) by immunocytochemistry. Nuclei were stained with DAPI. Scale bars, 20 μm. (**F**) HEI-OC1 cells were transfected with tfLC3 plasmids. After 18 h, the cells were treated with or without DOXO for 72 h, fixed, and analyzed by confocal microscopy. Decrease in autophagic reflux is indicated by the increased GFP/RFP ratio (blue arrowheads). Scale bars, 10 μm. (**G**) mTOC1 activity (p-P70S6K), lysosomal dysfunction, (LAMP1 and galectin-3), autophagic flux (LC3B) markers, and TFEB expression, were analyzed by immunoblotting in young and SIPS cells. β-Actin was used as the loading control. (**H**) qPCR analysis of TFEB target genes. *18S ribosomal RNA* was used as the control. Means ± SD. Experiments were performed in at least triplicate for each condition and repeated at least twice. # *p* < 0.05, *** *p* < 0.001 (Student’s *t*-test or one-way ANOVA, followed by Tukey’s HSD test).

The in vivo phenotype was investigated in a stress-induced premature senescence (SIPS) model of House Ear Institute organ of Corti 1 (HEI-OC1) cells [28]. When senescence was induced by low-concentration doxorubicin (DOXO), cells became enlarged (Figure 3A). Filipin staining was significantly increased (Figure 3B,C), the protein level and nuclear localization of TFEB were decreased, and lysosomal damage and autophagy were increased. These findings were in agreement with those obtained in vivo (Figure 3D–F and Supplementary Figure S4J). In a tandem mRFP-GFP-tfLC3B construct fluorescence analysis, RFP was more stable under acidic conditions, whereas GFP was rapidly quenched. Therefore, when autophagic flux was decreased, the GFP/RFP ratio was increased (Figure 3F). Moreover, the levels of the mature forms of lysosomal cathepsin B (CTSB) and cathepsin D (CTSD) increased during senescence but their activity significantly decreased (Supplementary Figure S4A–I), mTORC1 activity was upregulated (p-P70S6K), LAMP1, galectin-3, and LC3 I and II accumulated (Figure 3G), and the expression of TFEB and its target genes decreased (Figure 3G,H).

Therefore, the cochlear tissue phenotype in the ARHL group was similar to that of senescent cells.

### 2.4. Regulation of Lysosomal Biosynthesis and Autophagic Turnover by the mTORC1-TFEB Pathway during SIPS

We determined whether cholesterol regulates mTORC1 activity and the TFEB protein level. mTORC1 activity was induced by cholesterol-mediated P70S6K phosphorylation in HEI-OC1 cells (Supplementary Figure S5A). Cholesterol decreased the TFEB protein level and inhibited lysosomal and autophagic activity (Supplementary Figure S5B). This was confirmed by the modulation of the TFEB protein level by mTORC1 upon cholesterol treatment under mTORC1 inhibitor (Torin 1) and TFEB protein reduction (siTFEB) conditions (Supplementary Figure S5C,D).

**Figure 4:**
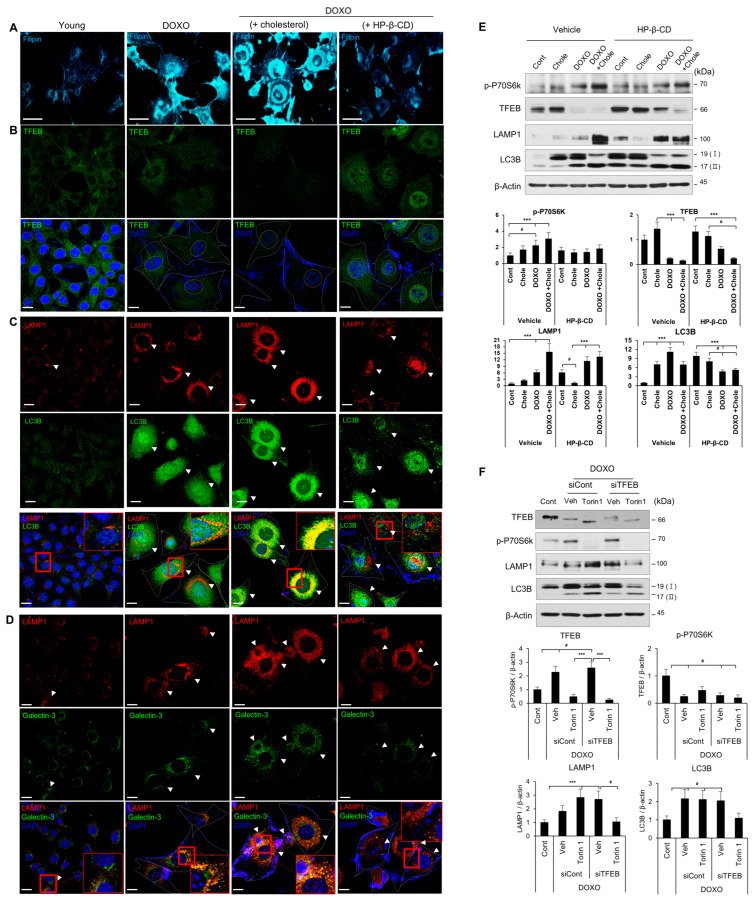
Inhibition of autophagic flux was accompanied by downregulation of TFEB via cholesterol-dependent mTORC1 activity in senescence. HEI-OC1 cells were treated with DOXO (100 ng/mL) for 24 h, treated with cholesterol (0.5 mM) or 2-hydroxypropyl-β-cyclodextrin (HP-β-CD, 6 mM), and maintained for 5 days. (**A**) Filipin staining of the cholesterol content of young and SIPS cells. Scale bars, 50 μm. (**B**–**D**) Immunolabeling using anti-TFEB, -LAMP1, -LC3B, and -galectin-3 antibodies to evaluate the relationship between autophagic flux inhibition and cholesterol with TFEB expression. Senescent cells show autolysosome accumulation (arrowheads, lanes 1–3) due to damaged lysosomes and high cholesterol. Cholesterol depletion (low cholesterol) reduces damaged lysosomes and autolysosomes (arrowheads, lane 4). DAPI was used as a counterstain. Red box shows co-localized proteins. Nuclei (yellow) and SIPS cells (white) are indicated by dotted lines. Scale bars, 20 μm; DAPI was used as the counterstain. (**E**) Cells were treated with or without DOXO (100 ng/mL) for 24 h and treated with cholesterol (0.1 mM) or HP-β-CD (6 mM) for 3 days. Only vehicle and cholesterol alone were used as the control. (**F**) Cells were transfected with siControl or siTFEB. After 24 h, the cells were treated with or without Torin 1 (0.5 μM) before administration of DOXO (100 ng/mL). Cells were maintained for 48 h, and the TFEB, p-P70S6K, LAMP1, and LC3B protein levels were analyzed by immunoblotting. β-Actin was used as the loading control. # *p* < 0.05, *** *p* < 0.001 (Student’s *t*-test or one-way ANOVA, followed by Tukey’s HSD test).

Cholesterol supply (high cholesterol) or depletion (low cholesterol; 2-hydroxypropyl-β-cyclodextrin [HP-β-CD]) conditions were used to determine whether cholesterol regulates mTORC1 activity and the TFEB protein level. Filipin staining was increased under high cholesterol conditions as compared to control senescent cells (Figure 4A, lanes 1–3), and TFEB expression and nuclear localization (Figure 4B, lanes 1–3) were reduced by mTORC1 activity, thereby suppressing the expression of target genes. Therefore, we observed the accumulation of autolysosomes through damaged lysosomes and high cholesterol levels in senescent cells (Figure 4C,D, lanes 1–3, Figure 4E and Supplementary Figure S5E). However, during cholesterol depletion (low cholesterol), filipin staining was similar to the control, and the accumulation of damaged lysosomes and autolysosomes was decreased (Figure 4A–D, lane 4, and Figure 4E). In addition, in normal cells treated with an mTORC1 inhibitor (Torin 1), the lysosomal and autophagy pathways were activated by increased TFEB activity and LAMP1 and LC3B (II) protein levels as compared to the control (Supplementary Figure S5C). However, upon TFEB knockdown, the TFEB, LAMP1, and LC3 I levels decreased, and mTORC1 was not inhibited (Supplementary Figure S5D). In addition, Torin 1-mediated inhibition of mTORC1 during cellular senescence increased TFEB activity and the LAMP1 and LC3B (I, II) levels. However, there was no change in LAMP1 and LC3B proteins upon TFEB knockdown (Figure 4F).

Therefore, cholesterol, which is abnormally increased during aging, induced mTORC1 activity and decreased TFEB activity, thereby inhibiting lysosomal biosynthesis and autophagy.

### 2.5. Atorvastatin Regulates Lysosomal Biosynthesis and Autophagic Activity by Decreasing mTORC1 Activity and Increasing TFEB Expression

**Figure 5:**
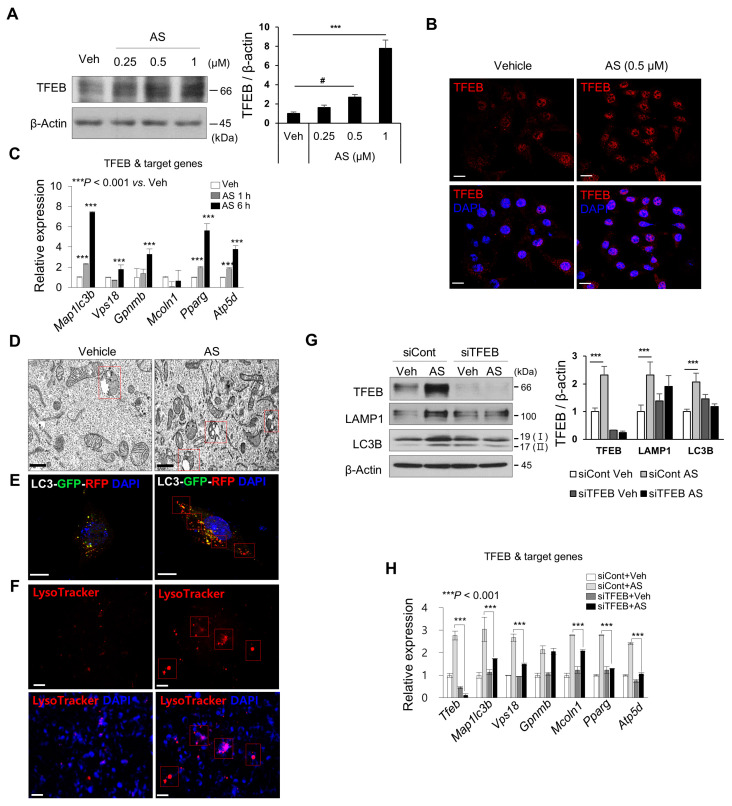
AS upregulates autophagy and lysosomal biogenesis by increasing the TFEB protein level and nuclear translocation. HEI-OC1 cells were treated with vehicle or AS (0.25 and 0.5 μM) for 6 h. (**A**) TFEB protein level by immunoblotting and (**B**) TFEB nuclear localization by immunocytochemistry. Scale bars, 50 μm. DAPI was used as the counterstain. (**C**) qPCR analysis of TFEB target genes at the indicated time points. *18S ribosomal RNA* was used as the control. (**D**) Autophagic vacuoles analyzed by TEM in vehicle- or AS-treated cells. Scale bars, 2 μm. Red boxes show autophagosomes or autolysosomes. (**E**) HEI-OC1 cells were transfected with tfLC3 plasmid for 24 h, treated with or without AS (0.5 μM) for 24 h, and analyzed by confocal microscopy. Scale bars, 20 μm. Red boxes show autophagosomes or autolysosomes. (**F**) HEI-OC1 cells were treated with vehicle or AS (0.5 μM) for 6 h and incubated with LysoTracker Red (50 nM) for 30 min. LysoTracker Red fluorescence was observed by fluorescence microscopy (Carl Zeiss MicroImaging GmbH). Scale bars, 20 μm. Red boxes show lysosomes. (**G**) To analyze autophagy- and lysosome-related protein levels, cells were transfected with siControl or siTFEB for 24 h, followed by AS (0.5 μM) for 6 h. (**H**) qPCR analysis of TFEB target genes. *18S ribosomal RNA* was used as the control. Means ± SD. Experiments were performed at least three times for each condition and repeated at least twice. # *p* < 0.05, *** *p* < 0.001 (Student’s *t*-test or one-way ANOVA, followed by Tukey’s HSD test).

Hydroxymethyl glutaryl coenzyme A reductase (HMG-CoA) inhibitors (i.e., statins) inhibit HMG-CoA, which is involved in the rate-limiting step of cholesterol biosynthesis [29]. Simvastatin maintains lysosomal biogenesis and autophagic turnover by regulating TFEB in mouse microvascular endothelial cells, thereby preventing cardiovascular complications such as endothelial barrier dysfunction with endothelial hyperpermeability in models of obesity [30]. A similar effect was confirmed in our pilot study. Upon Atorvastatin (AS) treatment of HEI-OC1 cells, the TFEB protein level (Figure 5A), nuclear localization (Figure 5B), and function (Figure 5C) were increased. Specifically, an increase in autophagic vacuoles was observed (Figure 5D), and an elevation in autophagic reflux, indicated by the increased red portion, was noted (Figure 5E). Additionally, lysosomal biosynthesis was confirmed to increase through lysotracker staining (Figure 5F). In addition, the LAMP1 and LC3B (I, II) protein levels and TFEB target gene expression were TFEB-dependently regulated in the presence of AS (Figure 5G,H). Therefore, lysosomal biogenesis and autophagic turnover were modulated by increased TFEB expression in AS-treated HEI-OC1 cells. 

**Figure 6:**
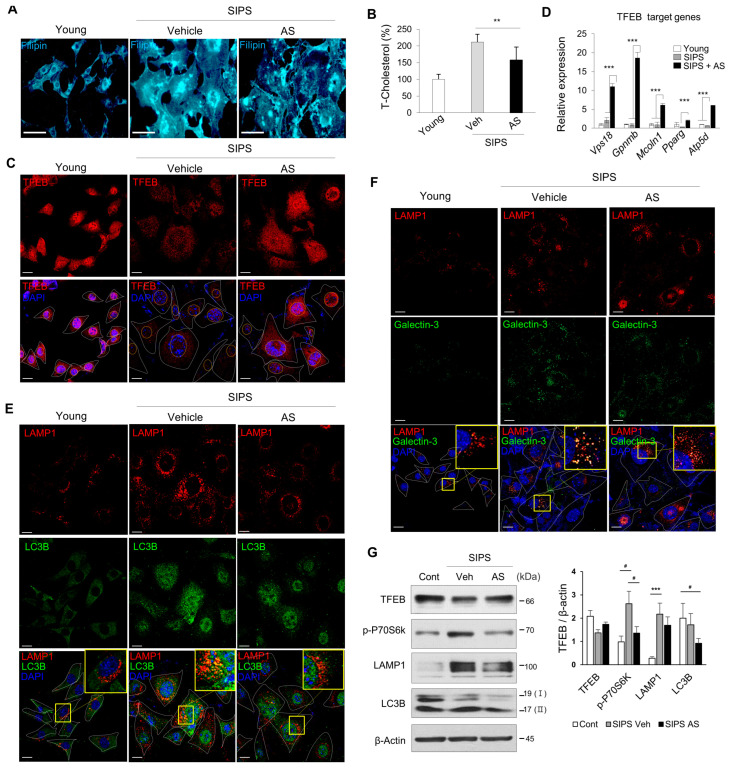
AS relieves damaged lysosome and autolysosome accumulation by decreasing cholesterol-mediated mTORC1 activity and increasing TFEB expression in SIPS. HEI-OC1 cells were treated with DOXO (100 ng/mL) for 24 h and treated with vehicle or AS (0.5 μM) for 3 days. (**A**) Filipin staining and (**B**) total cholesterol content of young and SIPS cells with or without AS. Scale bars, 50 μm. (**C**) Expression and nuclear localization of TFEB and (**E**) autophagy (LAMP1/LC3B)- and (**F**) lysosome (LAMP1/galectin-3)-related proteins by immunocytochemistry. Scale bars, 20 μm. DAPI was used as the counterstain. White or yellow dotted lines show cell bodies and nuclei, respectively. Yellow boxes show autolysosomes or damaged lysosomes. (**D**) qPCR of TFEB target genes. Means ± SD. *18S ribosomal RNA* was used as the control. (**G**) Confirmation of the immunofluorescence results by immunoblotting. Experiments were performed in at least triplicate for each condition and repeated at least twice. # *p* < 0.05, ** *p* < 0.01, *** *p* < 0.001 (one-way ANOVA followed by Tukey’s HSD test).

In SIPS cells treated with AS, cholesterol levels were significantly decreased as compared to the untreated control (Figure 6A,B), mTORC1 activity was decreased (Figure 6G), TFEB expression and nuclear localization were increased, and the expression levels of its target genes were increased (Figure 6C,D). Immunocytochemistry and immunoblotting revealed decreased LC3B/LAMP1 and LAMP1/galectin-3 accumulation by lysosome biogenesis and autophagic turnover (Figure 6E–G). 

### 2.6. AS Maintains TFEB-Mediated Lysosomal Biogenesis and Autophagic Turnover in Cochlear Tissues with ARHL

**Figure 7:**
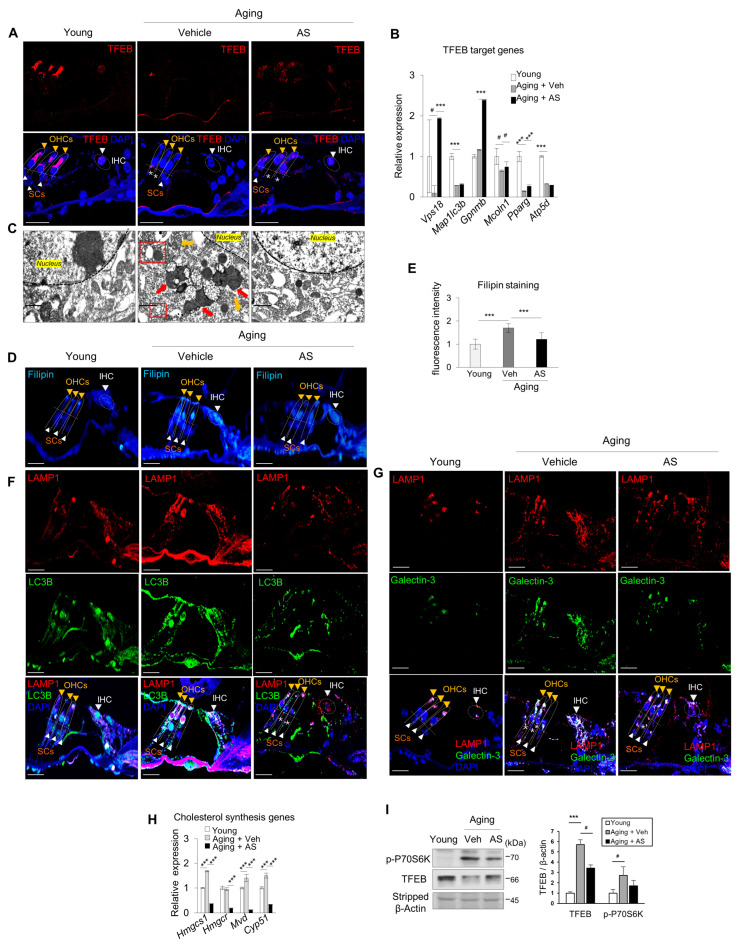
AS-treated ARHL mice preserved cochlear function by maintaining both cholesterol levels and autophagic flux. (**A**) Cochlear sections were immunolabeled using an anti-TFEB antibody and counterstained with DAPI. TFEB expression in IHCs, OHCs, and SCs of the young, aging-vehicle, and aging-AS groups (*n* = 3 for each group). Scale bars, 20 μm. (**B**) qPCR analysis of TFEB target genes. Means ± SD. *18S ribosomal RNA* was used as the control (*n* = 3 for each group). (**C**) Intracellular surface of hair cells observed by TEM in cochleae from young, aging-vehicle, and aging-AS mice. Red boxes show autolysosomes. Abnormally enlarged lipofuscin aggregates (red arrows) and mitochondria (yellow arrows) were frequently detected in the ARHL group as compared to the young group. Scale bar, 1 μm. (**D**) Filipin staining and (**E**) relative filipin fluorescence intensities were analyzed using ImageJ software (*n* = 3 replicates from two independent cochlea samples). (**F**) Levels of autophagy (LAMP1/LC3B)- and (**G**) lysosome (LAMP1/galectin-3)-related proteins confirmed by immunohistochemistry (*n* = 3 for each group). Scale bars, 20 μm. DAPI was used as the counterstain. White arrowheads indicate IHCs and SCs; yellow arrowheads indicate OHCs. Asterisks indicate cell loss. (**H**) qPCR of cholesterol synthesis-related gene expressions. *18S ribosomal RNA* was used as the control (*n* = 3 for each group). Means ± SD. (**I**) p-P70S6K and TFEB levels in whole cochlear fractions by immunoblotting. Experiments were performed at least three times for each condition and repeated at least twice. # *p* < 0.05, *** *p* < 0.001 (one-way ANOVA, followed by Tukey’s HSD test).

In c57BL/6N mice given a low concentration of AS, ARHL was significantly prevented [31]. In cochlear tissues with ARHL, TFEB was significantly decreased as compared to young tissues (Figure 7A), followed by a significant decrease in the expression of substrate genes (Figure 7B). Lipofuscin, abnormal lysosome, and autolysosome accumulation were also observed (Figure 7C). All of these phenotypes were reversible by administering AS. In addition, filipin staining and cholesterol synthesis were suppressed and autophagy inhibition and lysosomal damage were decreased by AS as compared to the control (Figure 7D–H). These findings are likely to have been caused by inhibition of mTORC1 activity and an increase in the TFEB protein level (Figure 7I). 

During aging, cholesterol accumulates because of abnormal production and is not sufficiently degraded due to decreased lysosomal function, inducing mTORC1 hyperactivity. This reduces the TFEB protein level and activity, suppressing lysosomal biogenesis and autophagic turnover. The result is a vicious cycle in which cholesterol, lipid lipofuscin, and damaged proteins accumulate and mTORC1 activity continues. This causes stress-mediated cell death in the inner ear (Supplementary Figure S1C–E). However, AS-mediated control of cholesterol levels could prevent hearing loss by inhibiting mTORC1 activity and maintaining the TFEB protein level and activity. This would regulate the balance between lysosomal biogenesis and autophagic turnover, thereby promoting cell survival.

## 3. Discussion

The changes in lipid and lipoprotein concentrations with aging increase the risk of atherosclerotic diseases such as cardiovascular diseases (CVD) [32]. Cardiovascular risk factors such as triglycerides, total serum cholesterol, LDL cholesterol, and HDL cholesterol are associated with hearing loss [33]. Therefore, statins may protect hearing by improving inner ear microcirculation [34]. Nevertheless, few studies have evaluated the relationship between hypercholesterolemia and inner ear pathology in the cochlea. The hearing preservation effects of statins are typically evaluated as secondary effects of suppressing CVD and improving cochlear microcirculation. Hypercholesterolemia affects hearing by modulating prestin synthesis in the OHC membrane microdomains of the organ of Corti, thereby reducing OHC electromobility [35,36,37]. The functions of guinea pig IHCs are regulated by potassium current changes, depending on the cholesterol level. Therefore, hypercholesterolemia may be linked to auditory function [38]. In this study, cholesterol levels and filipin staining were higher in ARHL cochlear tissues than in young tissues (Figure 1H and Figure 2B and Supplementary Figure S2), and abnormal OHC morphology and prestin synthesis were observed (Supplementary Figure S6). However, AS preserved hearing, decreased the cholesterol level (Figure 7D,E and Supplementary Figure S7), and prevented alteration of OHC formation by rearranging prestin (Supplementary Figure S6B) in the cochlea as compared to the control group.

Therefore, metabolic changes during aging result in an abnormally high cholesterol level in the cochlea. This suggests that cholesterol affects the organ of Corti, restricting cochlea microcirculation, in the development of ARHL. AS could preserve OHC function by maintaining prestin synthesis and controlling cholesterol levels during aging.

Autophagy degrades cellular components such as defective organelles and aggregates of unfolded, misfolded, and damaged proteins in lysosomes [39]. The levels of LC3 and its substrate p62 were increased in the cochlea of aged C57BL/6J mice, confirming impaired autophagy in ARHL mice [40]. Autophagy decreases with age, and its upregulation promotes aging hair cell (HC) survival and slows auditory cell degeneration [41,42,43,44]. Gentamicin, kanamycin, and furosemide induced HC loss and SGN degeneration by impairing autophagic flux and lysosomal biogenesis, and they restored autophagy by promoting SGN degeneration [22,45]. These findings suggest that autophagy activation by TFEB protects hearing by promoting the survival of cochlear hearing-sensing cells.

In this study, inhibition of autophagy was indicated by overlapping LAMP1 and LC3B expression and LC3 I and II accumulation (Figure 2E,G and Figure 3F,G). Lipofuscin accumulation in HCs and SGNs confirmed autophagy suppression (Figure 1B–D). However, autophagy was increased by TFEB activation when mTORC1 was inhibited by Torin 1 during senescence induction (Figure 4F). In addition, inhibition of hearing loss was confirmed by AS-induced autophagy in aging mice (Figure 7F) [31]. Therefore, maintaining autophagic activity during aging can protect hearing.

Age-associated dysregulation of autophagy (determined by the accumulation of autophagosomes), possibly due to impaired lysosomal fusion and/or degradation, is associated with cellular dysfunction and/or death, which contribute to neurodegeneration as well as cardiac and skeletal muscle aging [46,47,48,49,50]. Lysosomes exhibit age-related changes such as increased size, number, and content, while fluctuations in lysosomal hydrolase activity have also been reported [51,52,53,54,55,56]. However, the causal connection between age-associated lysosomal changes and abnormal lipid, cholesterol, and protein accumulation is unclear.

In lysosomal storage disorders (LSDs), genetic inactivation of lysosomal hydrolases or transporters triggers massive and pathogenic accumulation of their respective substrates within the lysosome [57,58]. Fabry disease is an X-linked hereditary lysosomal storage disease characterized by deficient activity of the lysosomal enzyme, alpha-galactosidase A (AGAL). Hearing loss is a feature of Fabry disease. In the NPC, a type of LSD, a genetic variation, disrupts lysosomal activity. This affects intracellular cholesterol transport and disturbs neurological phenotypes, including SNHL [2]. Also, lysosomal cholesterol-derived activation of mTORC1 inhibits autophagy and lysosomal biogenesis by inactivating TFEB [16].

In this study, the lysosomal marker, LAMP1, and the lysosomal damage and membrane permeability marker, cytosolic galectin-3 [59,60], co-localized in ARHL tissues but not in young tissues, suggesting lysosomal damage (Figure 2E). Abnormally shaped and larger lysosomes were frequently observed (Figure 1B–D and Figure 7C) with increased cholesterol and lipofuscin levels and mTORC1 activity and reduced TFEB protein level and autolysosome accumulation (LAMP1/LC3B) (Figure 2). These findings were recapitulated in the SIPS model (Figure 3). Lysosomal damage and abnormal accumulation of cholesterol were observed (Figure 4A,D and Figure 6A,F).

Lysosomal NPC1 expression (Supplementary Figure S3A,C) and lysosomal cathepsin B and D enzyme activities were decreased (Supplementary Figure S4A–I). Therefore, rather than being degraded, cholesterol and lipids accumulated in the form of lipofuscin. Cholesterol accumulation in lysosomes induces mTORC1 hyperactivity and reduces TFEB function, thereby suppressing autophagy and lysosome biogenesis-related gene transcription (Figure 4E,F and Supplementary Figure S5). Moreover, the levels of pro- and mature forms of CTSB and CTSD in mouse cochlear extract increased, but their activities decreased. This was similar to the effect of HEI-OC1 cellular senescence induction (Supplementary Figure S4A–I). There is evidence for increased lysosomal gene expression with age, which is likely to be a compensatory response to altered protein homeostasis [61]. Also, lysosomal damage increases membrane permeability, releasing enzymes such as CTSB and CTSD into the cytoplasm to activate caspase-mediated intrinsic apoptosis [62]. However, in this study, CTSB and CTSD activities decreased in the ARHL group as compared to the young group. Therefore, autophagy inhibition in ARHL tissue is linked to mTORC1-mediated decreased TFEB function via cholesterol accumulation and deterioration of lysosomal function.

TFEB regulates autophagic flux by promoting the expression of genes in the autophagy-lysosomal pathway. Overexpression of TFEB facilitates substrate clearance and alleviates the phenotypes associated with various diseases, such as Parkinson’s and Alzheimer’s diseases, in murine models by promoting autophagic flux [15,63]. mTORC1 activity increases during aging. Inhibition of the mTORC1 pathway extends lifespan in several models and protects against age-related pathologies. mTORC1 negatively regulates autophagy at multiple steps in its activation pathway [64]. mTORC1 inhibits autophagy by promoting nuclear export and cytoplasmic accumulation of TFEB by phosphorylating S142 and S138 at the nuclear export signal (NES) site [65]. Phosphorylated TFEB triggers proteasomal degradation via STUB1, a cytoplasmic chaperone-dependent ubiquitin ligase [66].

In this study, TFEB protein levels decreased in the in vivo and in vitro models of aging (Figure 2I and Figure 3D,G). In addition, the protein level in the cytoplasm decreased after nuclear export during senescence (Supplementary Figure S4J). This is probably because TFEB in the cytoplasm is degraded in a proteasome-dependent manner after phosphorylation by mTORC1, which is activated during aging. c-Abl induces TFEB tyrosine phosphorylation, inhibiting TFEB-induced cholesterol clearance [67]. 

Mitochondrial biogenesis is tightly regulated by several transcription coactivators and nuclear transcription factors, such as peroxisome proliferator-activated receptor gamma (PPARγ) coactivator-1alpha (PGC-1α), nuclear respiratory factor 1 (NRF1), nuclear respiratory factor 2 (NRF2), and mitochondrial transcription factor A (TFAM) [68]. PGC-1α inhibition reduces TFEB activity and disturbs the expression of several genes directly involved in mitochondrial biogenesis and function [69]. Overexpression of TFEB in mice increased the expression of NRF1, NRF2, and TFAM [70]. TFEB influences the levels of mitochondrial proteins COX I, COX II, COX IV, ATP5A1, and cytochrome c, enhancing respiratory chain activity [70,71,72].

In this study, TFAM and the expression of dihydroethidium (DHE), a redox-sensitive probe, were increased in ARHL tissues (Supplementary Figure S8A, top and middle lines). In addition, damaged and abnormal mitochondria were observed at a high frequency (Supplementary Figure S8A, bottom line), and the mtDNA content was increased (Supplementary Figure S8B). Mitochondrial reactive oxygen species (ROS) levels were increased by the accumulation of dysfunctional mitochondria in ARHL tissues. In the in vitro cell model, mtDNA accumulated, the mitochondrion-related ROS level increased (MitoSox), adenosine triphosphate (ATP) generation decreased, and TFAM expression decreased, whereas the number of mitochondria increased (Supplementary Figure S8F–H). However, mitochondrial biogenesis and ATP production increased because of upregulated expression of mitochondrion-encoded genes and TFAM in the AS-treated group in vivo and in vitro (Supplementary Figure S8C,E,H). In addition, increased autophagy (mitophagy) decreased the ROS level by reducing the number of dysfunctional mitochondria (Supplementary Figure S8A,B,F,G). Therefore, the balance of energy metabolism in auditory cells was maintained by replacing damaged mitochondria.

The limitation of the study is that the data in this experiment are based on a mouse model. Therefore, the pathogenesis in the human inner ear appears to require additional experiments. This necessitates further investigation through experiments involving human samples or human inner ear organoids. To explore the translational relevance of these discoveries, it is essential to conduct further prospective, multicenter, double-blind studies with individuals experiencing ARHL. These studies will specifically investigate the inhibition of autophagy caused by abnormally high cholesterol levels, the decreased lysosomal function during the aging process, and the impact of atorvastatin on ARHL patients.

**Figure 8:**
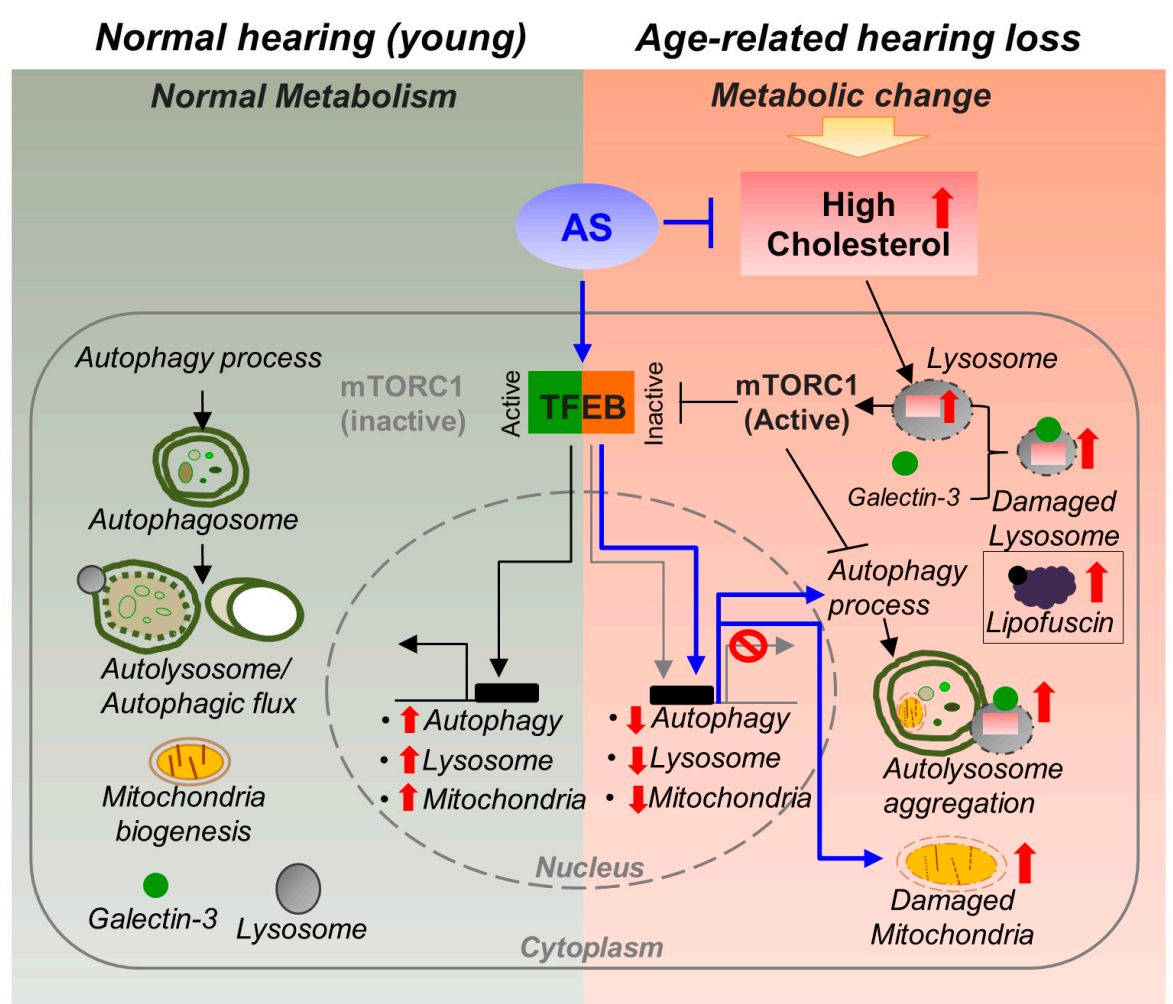
Cholesterol-mediated ARHL pathogenesis and the inhibitory effect of AS. Aging-related metabolic changes result in an abnormally high cochlear cholesterol level. Aging-related lysosomal damage leads to accumulation of damage proteins, lipids, and cholesterol. Accumulated cholesterol in lysosomes promotes mTORC1 activity, leading to protein inactivation and TFEB reduction (which regulates lysosomal and mitochondrial biosynthesis). mTORC1 activity inhibits autophagy, increasing dysfunctional mitochondria accumulation and ROS production, leading to increased intracellular stress. However, AS suppresses cholesterol synthesis, accumulation, and mTORC1 activity, and increases the level and activity of TFEB, thereby activating autophagy to clear damaged proteins and organelles, thus reducing intracellular ROS and stress. In addition, the ATP level is increased as a result of increased mitochondrial biosynthesis, maintaining intracellular homeostasis. As a result, AS maintains vascular health by reducing the cholesterol level, improving CVD and inner ear microcirculation, and maintains autophagic flux through TFEB protein expression and functional activity in the cochlea and hearing through protection of mitochondrial function.

In conclusion, the pathogenesis of ARHL involves the suppression of autophagy by an abnormally high cholesterol level and decreased lysosomal function during aging. However, atorvastatin reduces cholesterol and mTORC1 activity, suggesting that hearing loss could be prevented by maintaining lysosomal biosynthesis, autophagy, and mitochondrial function (Figure 8). This research has important implications for understanding and potentially addressing ARHL. It identifies cholesterol as a risk factor for ARHL and emphasizes the unclear impact of cholesterol on the organ of Corti during its onset. The study’s future prospects indicate that positive results in clinical trials could help to reduce the social and economic challenges linked to ARHL, especially in an aging population.

## 4. Materials and Methods

### 4.1. Animals

Age-matched male CBA/J mice (6 months of age) were obtained from Dae Han Bio Link Co., Ltd. (Chungbuk, Republic of Korea). All procedures involving animals were approved by the Institutional Animal Care and Use Committee of Ajou University Graduate School of Medicine, Suwon, Republic of Korea (2021–0011). The animals were maintained under standard animal house conditions. We established age-related hearing loss in mice at 24 months. Auditory thresholds were measured in terms of the ABR before and after 24 months of age. Mice at 6 months of age were used as young controls. After confirming ARHL, mice were euthanized.

### 4.2. Auditory Brainstem Response Analysis

Mice were anesthetized by intraperitoneal injection of Zoletil^®^ 50 (tiletamine-zolazepam, 40 mg/kg; Virbac Animal Health, Carros, France) mixed with Rompun^®^ (xylazine, 10 mg/kg; Bayer Health Care, Seoul, Republic of Korea). The core temperature was maintained at 37 °C using a heating pad. For hearing threshold evaluation, needle electrodes were inserted subcutaneously at the vertex, under the pinna of the left ear and the right ear. ABRs were measured at frequencies of 8, 16, and 32 kHz with tone-burst stimuli reducing levels at 10–80 dB in a soundproof booth. The ABR threshold was defined as the lowest stimulus level at which a clear waveform was visible in the evoked trace using the TDT II System and BioSig software (version 5.7.5, Tucker Davis Technologies, MathWorks, Naples, FL, USA).

### 4.3. Cell Culture and Doxorubicin Treatment

HEI-OC1 cells were cultured at 37 °C and 5% CO_2_ in high-glucose Dulbecco’s Modified Eagle’s Medium (11965092, Gibco; Thermo Fisher Scientific, Inc., Waltham, MA, USA) containing 10% fetal bovine serum (16000044, Gibco) without antibiotics. The cells were seeded in the desired numbers and exposed to DOXO (100 ng/mL) for 5 days. The cells were maintained in complete medium, which was changed at 3 days. DOXO-induced cellular senescence was monitored by assaying the expression of p53 and p21WAF1/Cip at the indicated times. Senescence morphology was captured by bright-field microscopy (Olympus BX-60, Tokyo, Japan).

### 4.4. Immunoblotting

Whole cochlea tissues were prepared by polytron homogenization followed by extraction in radioimmunoprecipitation assay (RIPA) buffer containing 50 mM Tris/HCl (pH 7.5), 150 mM NaCl, 1.0% nonidet P40, 0.1% sodium dodecyl sulphate (SDS), 0.5% deoxycholic acid, 1.0 μg/mL leupeptin, 100 μg/mL phenylmethylsulfonyl fluoride (PMSF), 1.0 mM Na3VO4, and 1.0 mM NaF. Harvested cells were solubilized in RIPA buffer and cleared by centrifugation at 12,000× *g* for 20 min at 4 °C, and protein concentration was determined using a DC Protein Assay Kit II (5000112, Bio-Rad, Hercules, CA, USA). Lysate (20 μg per lane) was resolved by 10–15% SDS polyacrylamide gel electrophoresis (SDS-PAGE) in 25 mM Tris/glycine buffer. Protein bands were transferred to polyvinylidene fluoride (PVDF) membranes, incubated with 5% non-fat skim milk in phosphate-buffered saline (PBS, 21-040-CMX12) containing 0.05% Tween 20 (PBST) for 1 h, and then incubated with the appropriate antibodies overnight at 4 °C. The PVDF membranes were washed three times with PBST and incubated with horseradish peroxidase-conjugated secondary antibodies for 1 h. An enhanced chemiluminescence (ECL; Amersham Biosciences, Little Chalfont, UK) kit was used to evaluate protein levels. Band intensity was analyzed using ImageJ software (version 1.54g, National Institutes of Health, Bethesda, MD, USA)

### 4.5. Hematoxylin and Eosin and Immunohistochemical Staining

Cochleae were dissected, fixed with 4% paraformaldehyde, and decalcified in Calci-Clear Rapid Decalcifying Solution (HS-105, National Diagnostics) for 4 days. Decalcified cochleae were embedded in paraffin. For immunofluorescence analysis, paraffin-embedded sections were cut at 6 μm thickness. Cochlear sections were dewaxed in xylene, rehydrated in a series of graded ethanol washes (100%, 90%, 80%, and 70%), and subjected to histological analysis by hematoxylin and eosin (H&E) staining. For antigen retrieval, slides were washed several times in distilled water. Next, two to four drops of pepsin solution (750102, Invitrogen Ltd., Paisley, UK) were added and the sections were incubated for 30 min at 37 °C. The cochlear sections were washed in distilled water and endogenous peroxidase was blocked with 3% hydrogen peroxide (7722-84-1, Sigma-Aldrich, St. Louis, MO, USA) for 15 min. Cochlear sections were incubated for 1 h at room temperature in blocking/permeabilization solution containing 3% bovine serum albumin (A0100-005, GenDEPOT, Barker, TX, USA) and Triton X-100 (0.05%) in 0.1 M PBS. Next, the sections were incubated with primary antibodies overnight at 4 °C. After three washes with 0.1 M PBS, the sections were incubated at room temperature for 1 h with the appropriate secondary antibodies. We performed counterstaining with 4′,6-diamidino-2-phenylindole (DAPI). For the negative controls, primary antibodies were substituted with nonimmune sera. Sections were visualized under a confocal microscope (Carl Zeiss MicroImaging GmbH, Jena, Germany).

### 4.6. Cholesterol Content

The total cholesterol content in whole cochlear extracts and cell lysates was measured using the Cholesterol/Cholesteryl Ester Quantitation Assay Kit (ab126287, Abcam, Cambridge, MA, USA), according to the manufacturer’s instructions.

### 4.7. Filipin Staining

To assay membrane cholesterol content, we used the fluorescent probe, filipin, which binds to membrane cholesterol [73]. Briefly, frozen sections of fixed tissue or fixed cells (8 × 10^4^ on P35 dishes) were stained with filipin III solution (0.5 mg/mL) for 30 min. Next, samples were washed three times with PBS and immediately examined under an immunofluorescence microscope (Olympus, Tokyo, Japan). Fluorescence intensity was quantified based on pixel intensity/area using ImageJ software (version 1.54g, National Institutes of Health, Bethesda, MD, USA). The OCs at the mid-cochlear turn were selected as the region of interest (ROI) for filipin fluorescence. Using the freehand selection tool, we selected filipin-positive stained ROIs and calculated the pixel intensity/area.

### 4.8. Electron Microscopy

Cochlear tissues were post-fixed in 1% osmium tetroxide, dehydrated in 70–100% ethanol, incubated in propylene oxide, and embedded in Embed 812 resin (14900, Electronic Microscopic Science, Hatfield, PA, USA). Cochlea histology was observed under an EM902A microscope (Carl Zeiss MicroImaging, Oberkochen, Germany) at the specified magnification.

### 4.9. Real-Time PCR

Total cellular RNA was extracted using RNAiso Plus (9108, TaKaRa, Shiga, Japan), and cDNA was synthesized from 1.0 μg of RNA using the PrimeScript 1st strand cDNA Synthesis Kit (6110A, TaKaRa, Shiga, Japan). cDNA was amplified in a CFX96 Real-Time PCR Cycler (Bio-Rad) using specific primers and SYBR Green PCR Master Mix (NanoHelix Co., Ltd., Daejeon, Republic of Korea) under the following conditions: activation at 95 °C for 15 min, followed by 35 cycles of 95 °C for 20 s and 60 °C for 30 s. The primers are listed in Supplementary Table S1.

### 4.10. siRNA Transfection

Control siRNAs (sc-37007) and siRNA against TFEB (sc-38510) were purchased from Santa Cruz Biotechnology (Dallas, TX, USA). HEI-OC1 cells (1 × 10^6^/well) were transfected with siRNAs and Lipofectamine RNAiMAX Transfection Reagent (13778075, Invitrogen, Carlsbad, CA, USA) for 48 h, and DOXO was administered at the indicated time points. The cells were subjected to immunoblotting or qPCR.

### 4.11. Plasmid Transfection

HEI-OC1 cells (5 × 10^4^/well) on coverslips in 6-well plates were transiently transfected with 1 μg of plasmid DNA using Lipofectamin3000 reagent (Life Technologies, Carlsbad, CA, USA) and incubated for 48 h. The cells were treated with or without AS (0.25 μM) for the indicated times and subjected to immunocytochemistry. mRFP-GFP tandem fluorescence-tagged LC3B (tfLC3B) was provided by Dr. T. Yoshimori (Osaka University, [74]).

### 4.12. RNA Sequencing

Total RNA was extracted from the cochleae of CBA/J mice (young = 3, ARHL = 3, respectively) using the RNAprep Pure Kit for Tissue (4992236, Tiangen Biotech, Beijing, China), according to the manufacturer’s instructions. RNA quality and quantity were assessed using a Colibri Microvolume Spectrophotometer (Titertek-Berthold, Pforzheim, Germany). RNA library preparation and RNA sequencing were performed by Macrogen, Inc. (Seoul, Republic of Korea) using the Illumina HiSeq4000 Sequencing Platform (Illumina, San Diego, CA, USA).

### 4.13. Gene Enrichment and Functional Annotation Analyses

Differentially expressed transcripts were identified using Cuffdiff software (http://cole-trapnell-lab.github.io). Transcripts with |FC| ≥ 2 and raw *p* < 0.05 were defined as differentially expressed genes (DEGs). Gene ontology (GO) enrichment analysis of the DEGs was conducted using the g:Profiler tool (https://biit.cs.ut.ee/gprofiler/). GO terms with an adjusted *p* < 0.05 were considered significantly enriched. The analysis was performed by Macrogen, Inc.

### 4.14. Gene Set Enrichment Analysis

A ranked list was generated based on the FC and raw *p*-values between CBA/J 24 M and CBA/J 6 M. We used the Java program, GSEA-P 4.0.3, which implements the GSEA algorithm to identify genes showing statistically significant differences (http://www.broadinstitute.org/gsea). Analysis of biological function using the BIOCARTA, KEGG, and Gene Ontology databases was performed using the MSigDB gene set database.

### 4.15. Ingenuity Pathway Analysis

Network interactions among top disease and biological function pathways were evaluated using Ingenuity Pathway Analysis software, version 60467501 (Ingenuity systems, Redwood City, CA, USA) based on the DEG data. Significantly upregulated disease and disorder-related genes are shown.

### 4.16. Antibodies and Reagents

Phospho-mTOR (Ser2481) antibody (2974), phospho-p70 S6 kinase (Thr389) antibody (97596), and phospho-4E-BP1 (Thr37/46) antibody (2855) were obtained from Cell Signaling Technology; galectin-3 (ab2785) and LAMP1 (ab24170; for immunoblot) were obtained from Abcam; LC3B (sc-16755) and LAMP1 (sc-19992; for IHC) were obtained from Santa Cruz Biotechnology; TFEB (13372-1-AP) was obtained from Proteintech. LC3B (L7543), filipin (SAE0087), doxorubicin (D1515), hydroxypropyl-β-cyclodextrin (HP-β-CD) (C0926), cholesterol (C4951), SBB (199664), and Nuclear Fast Red (NFR) (ab246831) were purchased from Sigma-Aldrich.

### 4.17. Statistical Analysis

Data are shown as means ± standard deviation (SD) or standard error of the mean (SEM) of at least three independent experiments. Differences among means were assessed using Student’s *t*-test for two groups and one-way analysis of variance (ANOVA) for multiple groups followed by Tukey’s honestly significant difference (HSD) test in SPSS software (version 23.0, IBM, Armonk, NY, USA). Significance was evaluated at a level of *p* < 0.05.

## Data Availability

All data presented in this study are available upon reasonable request from the corresponding author.

## Supplementary Materials

The following supporting information can be downloaded at: https://www.mdpi.com/article/10.3390/ijms242417513/s1. References [75,76,77] are cited in the Supplementary Materials.
