# Supplementary material for: Abnormal Cholesterol Metabolism and Lysosomal Dysfunction Induce Age-Related Hearing Loss by Inhibiting mTORC1-TFEB-Dependent Autophagy
Source: Int J Mol Sci. 2023 Dec 15;24(24):17513. doi: 10.3390/ijms242417513 (PMC10743727; doi:10.3390/ijms242417513)
Supplement: Supplementary file 1 [file ijms-24-17513-s001.zip › ijms-2749027-supplementary.pdf]

Table S1. Real-time PCR primer list

Target gene	Primer sequences
Tfeb-F	CAG CAG GTG GTG AAG CAA GAG T
Tfeb-R	TCC AGG TGA TGG AAC GGA GAC T
Map1lc3b-F	TTC TTC CTC CTG GTG AAT GG
Map1lc3b-R	GTG GGT GCC TAC GTT CTC AT
Vps18-F	TTG TCG TCT CCA GCA ATC AG
Vps18-R	CCT TGC CCA AGT CAA TGC
Gpnmb-F	AGA AAT GGA GCT TTG TCT ACG TC
Gpnmb-R	CTT CGA GAT GGG AAT GTA TGC C
Mcoln1-F	CTG ATG CTG CAA GTG GTC AA
Mcoln1-R	GGT GTT CTC TTC CCG GAA TGT C
Pparg-F	GTG ATG GAA GAC CAC TCG CAT T
Pparg-R	CCA TGA GGG AGT TAG AAG GTT C
Atp5d-F	CAT GTC CCC ACA CTA CAG GTC
Atp5d-R	TCG GCA TTC ACA GTG ACG
Hmgcs1-F	TGA TCC CCT TTG GTG GCT GA
Hmgcs1-R	AGG GCA ACG ATT CCC ACA TC
Hmgcr-F	ATC CTG ACG ATA ACG CGG TG
Hmgcr-R	AAG AGG CCA GCA ATA CCC AG
Mvd-F	CTG CAC CAG GAC CAG CTA AA
Mvd-R	CTG AGG CTG AGG GGT AGA G
Cyp51-F	GTT TCA GGC GCA GGG ATA GA
Cyp51-R	CAT CTG TTA GAG GAC GCC CG

Nd1-F	CCC ATT CGC GTT ATT CTT
Nd1-R	AAG TTG ATC GTA ACG GAA GC
Sdhb-F	TGG TGG AAC GGA GAC AAG TA
Sdhb-R	TGG CAG CGG TAG ACA GAG AA
Uqcrc1-F	GGG GCA AAA ACA TCC TTA GG
Uqcrc1-R	ATC CGG CTC TCC CAC TCA GC
Cox5b-F	CGT CCA TCA GCA ACA AGA GA
Cox5b-R	AGA TAA CAC AGG GGC TCA GT
Atp6-F	TCC CAA TCG TTG TAG CCA TCA
Atp6-R	AGA CGG TTG TTG ATT AGG CG
Npc1-F	TGA GGT CAC GGG GCC ATG TA
Npc1-R	GGC ATC CAA GCC CCA GAT CC
Npc2-F	TAT CTT GTG ACT GCT CGG
Npc2-R	CTG GTA AAG GTG ATG TTG A
Srebp-1-F	AAG CAA ATC ACT GAA GGA CCT GG
Srebp-1-R	AAA GAC AAG GGG CTA CTC TGG GAG
Srebp-1c-F	CTT CTG GAG ACA TCG CAA AC
Srebp-1c-R	GGT AGA CAA CAG CCG CAT C
Srebp2-F	GCG TTC TGG AGA CCA TGG A
Srebp2-R	ACA AAG TTG CTC TGA AAA CAA ATC A
Fasn-F	CAG CAG AGT CTA CAG CTA CCT
Fasn-R	ACC ACC AGA GAC CGT TAT GC
Scd1-F	AGA GAG AGA GGT AGC CAT ATC
Scd1-R	TCA AAT CTC ACT AAT CTC TGG

18S rRNA -F	GTA ACC CGT TGA ACC CCA TT
18S rRNA -R	CCA TCC AAT CGG TAG TAG CG

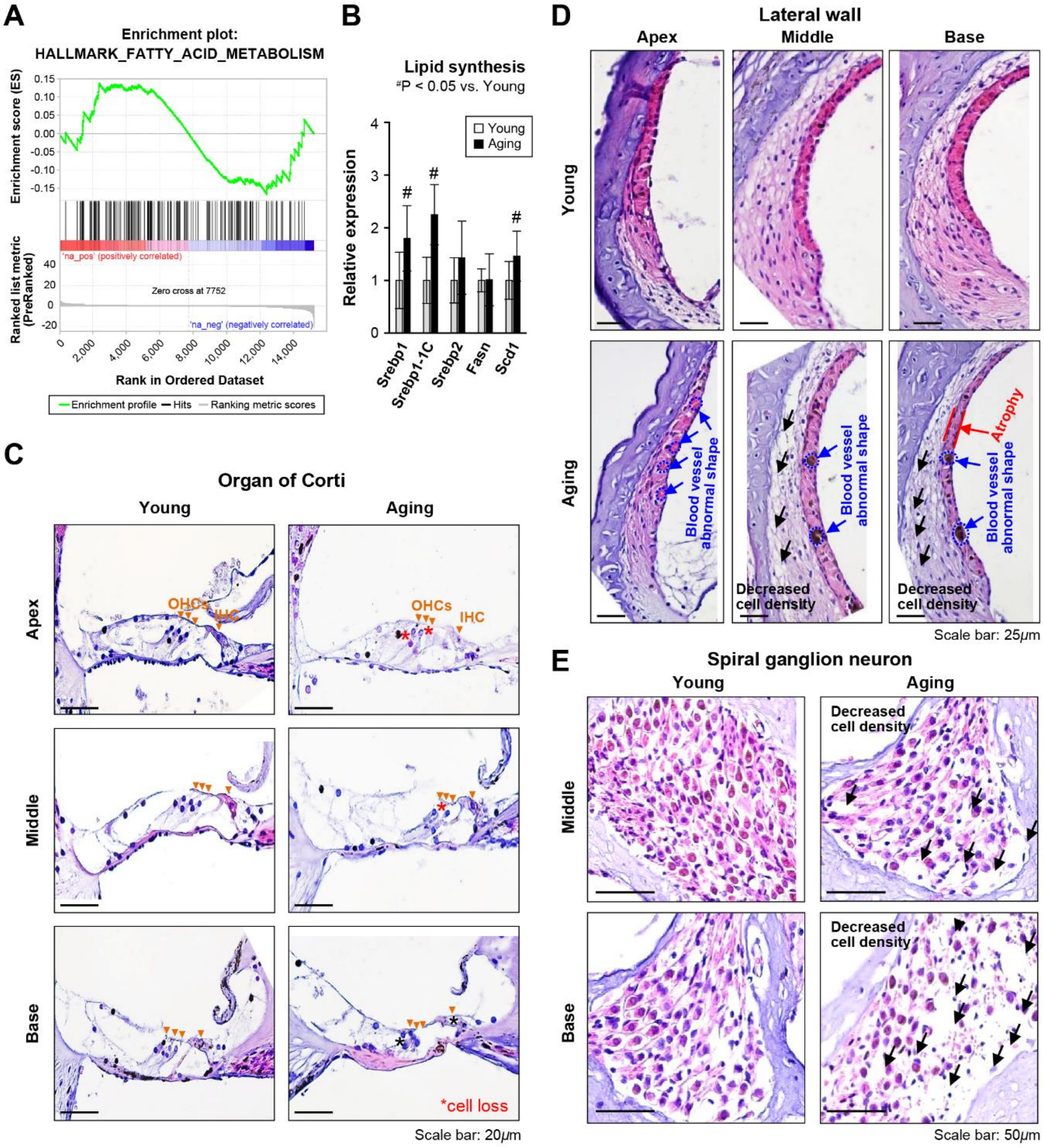

Figure S1. Histological analysis of the organ of Corti, striavascularis at the lateral wall, and spiral ganglion neurons of the cochlear tissue. (**A**) Gene set enrichment assay (GSEA) showed that genes whose expression was positively correlated with fatty acid metabolism were upregulated in the age-related hearing loss (ARHL), as compared to the young group. (**B**) qPCR analysis of lipid synthesis-related genes. Means ± standard deviation (SD). (**C**) Hematoxylin and eosin (H&E)-stained sections from the cochlear apex to the basal turn. Arrowheads indicate

inner hair cells (IHCs) and outer hair cells (OHCs). Asterisks indicate cell loss. (**D**) Abnormal blood vessel shapes, (**C–E**) decreased cell density, and (**C**) striavascularis atrophy are indicated by blue, black, and red arrows, respectively. Experiments were performed in at least duplicate #p < 0.05 (Student's *t*-test).

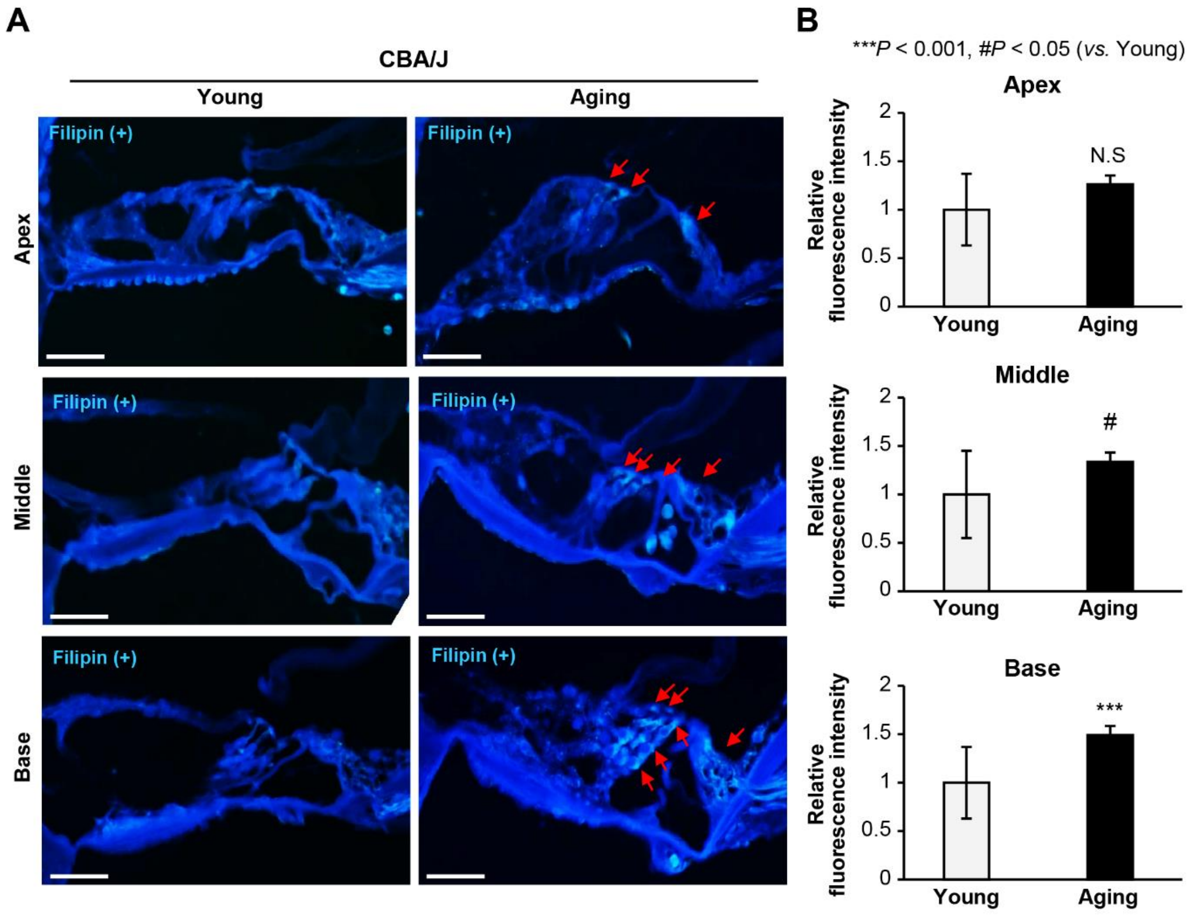

Figure S2. ARHL cochleae have an increased cholesterol level in the organ of Corti. (**A**) Filipin staining from the cochlear apex to the basal in young and aging mice. Arrows indicate filipin-positive IHCs, OHCs, and supporting cells (SCs). Scale bars, 20 µm. (**B**) Filipin fluorescence intensity in young (n = 4) and aging (n = 4) tissue sections. Means ± standard error of the mean (SEM). #p < 0.05, ***p < 0.001 by Student's *t*-test.

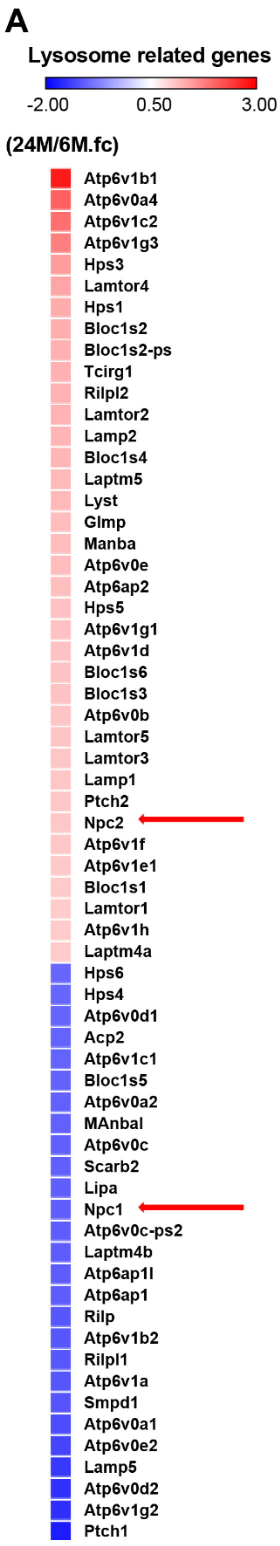

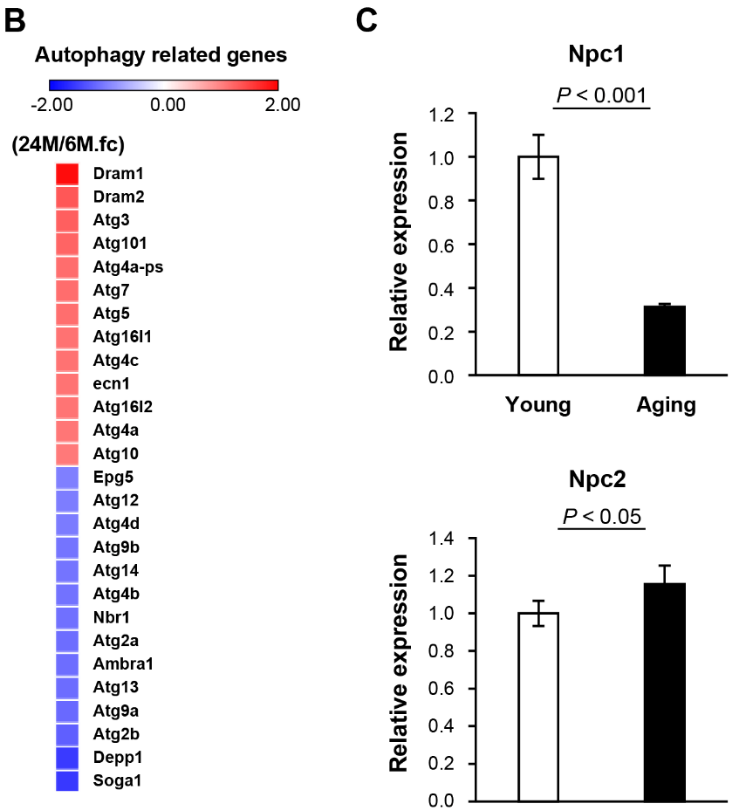

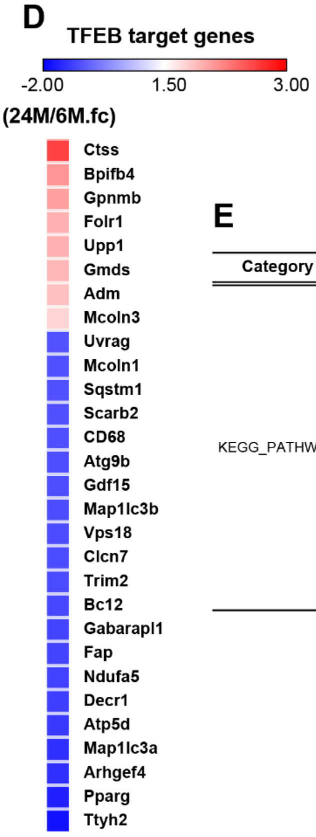

E

Category	Term	Gene count	%	P-Value	Benjamini
KEGG_PATHWAY	Autophagy-animal	7	24.1	6.40E-07	5.30E-05
	Mitophagy-animal	5	17.2	1.70E-05	6.80E-04
	Amyotrophic lateral sclerosis	6	20.7	1.40E-03	3.70E-02
	Lysosome	4	13.8	3.70E-03	6.60E-02
	Pathways of neurodegeneration-Multiple diseases	6	20.7	4.00E-03	6.60E-02
	NOD-like receptor signaling pathway	4	13.8	1.30E-02	1.70E-01
	Autophagy-other	2	6.9	7.30E-02	8.50E-01
	Ferroptosis	2	6.9	9.00E-02	9.20E-01

Figure S3. Heat map of transcription factor EB (TFEB) and differentially expressed genes

(DEGs) related to the lysosome and autophagy. (**A and B**) Autophagy and lysosome-related heatmap results analyzed through functional annotations of lysosomes, including Niemann–Pick C1 (NPC1) and 2 (indicated by red arrows), as well as autophagy from DEG raw data. (**C**) Validation of *Npc1* and *Npc2* by qPCR in the ARHL, as compared to the young group. Means ± SD. (**D**) TFEB target gene heat map result was analyzed from DEG raw data according to published articles [1-3]. For KEGG pathway enrichment analysis, we used the web-based DAVID tool (https://David.ncifcrf.gov/home.jsp). (**E**) KEGG pathway enrichment analysis of DEGs with autophagy, the lysosome, and autophagy-related diseases. Experiments were performed at least three times for each condition and repeated at least twice $p < 0.05$, $p < 0.001$ (Student's *t*-test). DEG raw data are presented in the supplemental source file. Heat map was prepared using MORPHEUS online software (https://software.broadinstitute.org/morpheus/).

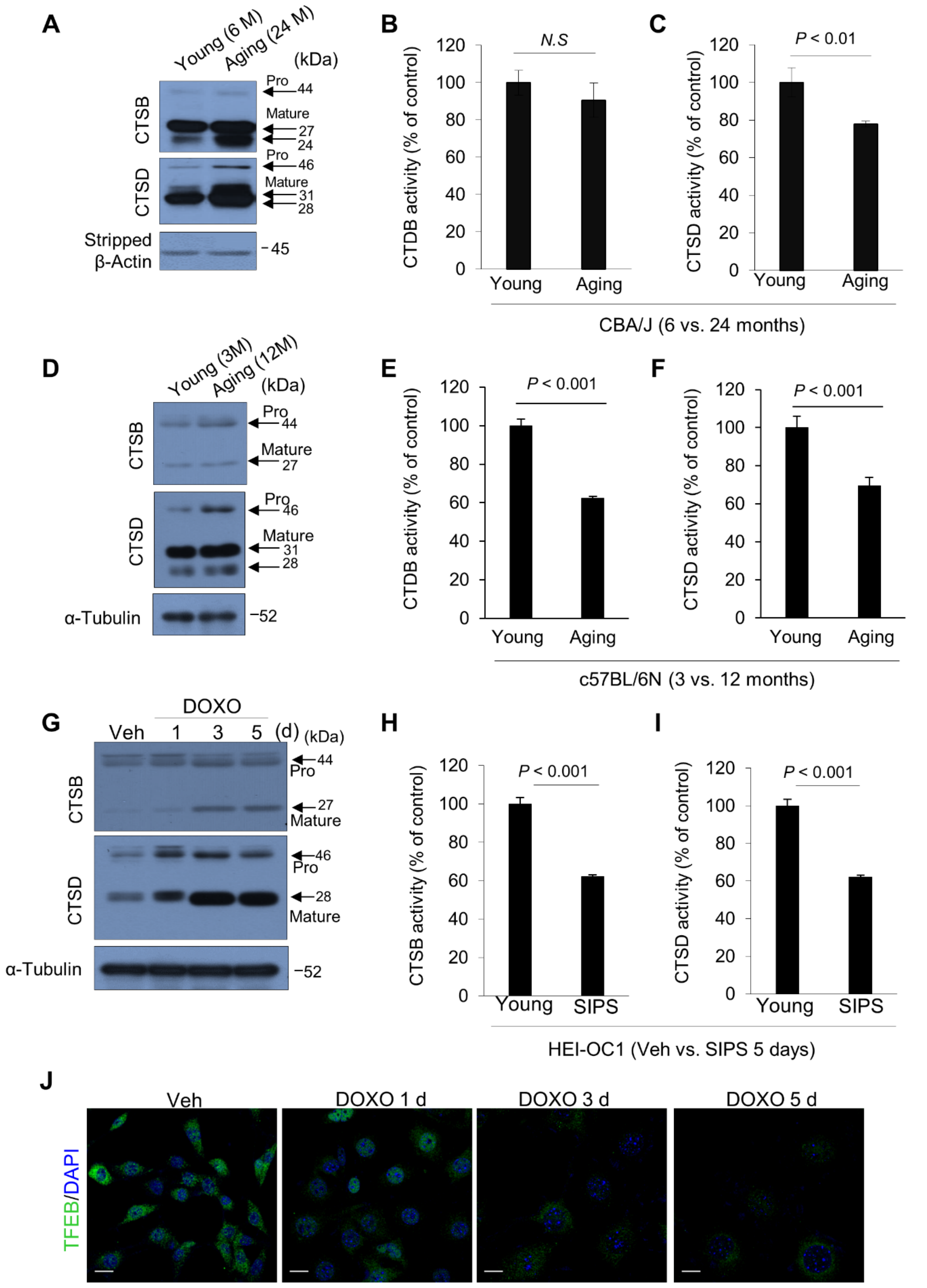

Figure S4. Cathepsin B, D (CTSB, D) expression and activities in two mouse models and

models of SIPS. (**A, D and G**) CTSB, CTSD protein expression and (**B, C, E, F, H and I**) CTSB, CTSD activities were measured in whole cochlea or cell extract by immunoblotting and using an enzyme activity assay kit. β-Actin or α-tubulin were used as the loading control for immunoblotting. Means ± SD. (**J**) TFEB expression and nuclear localization by immunocytochemistry with or without doxorubicin (DOXO; 100 ng/mL). Nuclei were stained with 4′,6-diamidino-2-phenylindole (DAPI). Scale bars, 20 μm. Experiments were performed at least three times for each condition and repeated at least twice; $p < 0.01$, $p < 0.001$ (Student's *t*-test).

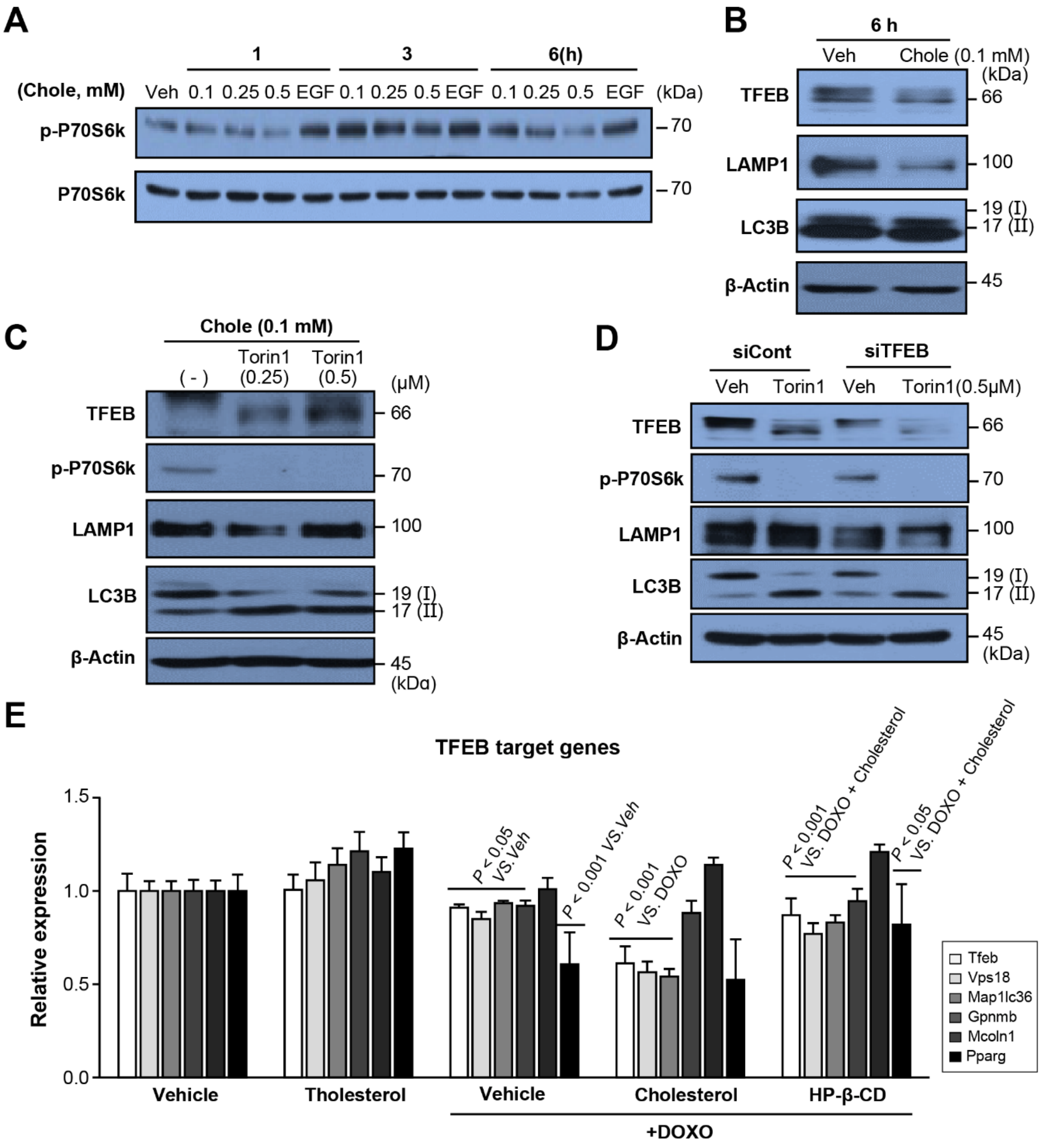

Figure S5. Cholesterol negatively regulates TFEB expression by increasing mammalian target of rapamycin complex 1 (mTORC1) activity. (**A**) House Ear Institute organ of Corti 1 (HEI-OC1) cells were treated with cholesterol (0.1, 0.25, and 0.5 mM) for 1, 3 and 6 h. mTORC1 activity was assayed by measuring phosphorylation of p70S6K. p70S6K was used as the loading control. Epidermal growth factor (EGF; 10 ng/mL) was used as the positive control. Cells were treated with cholesterol (0.1 mM) for 30 min, and Torin1 (0.5 μM) for 6 h. (**B**)

TFEB, LAMP-1, and LB3B protein levels were downregulated by cholesterol at 6 h; the effect was reversed (**C**) by an mTORC1 inhibitor (Torin 1). β-Actin was used as the loading control. (**D**) To analyze whether autophagy- and lysosome-related protein expressions are regulated by TFEB protein, cells were transfected with siControl or siTFEB. After 24 h, the cells were treated with or without Torin1 (0.5 μM) for 48 h. (**E**) To analyze TFEB target gene expression under high or low cholesterol conditions in the presence of DOXO (100 ng/mL) for 24 h, the cells were treated with cholesterol (0.1 mM) or 2-hydroxypropyl-β-cyclodextrin (HP-β-CD; 5 mM) for 3 days. (**D**) qPCR analysis of TFEB target genes under identical conditions. *18S ribosomal RNA* was used as the control. Means ± SD. Experiments were performed at least three times for each condition and repeated at least twice. $p < 0.05$, $p < 0.001$ (one-way analysis of variance [ANOVA] followed by Tukey's honestly significant difference [HSD] test).

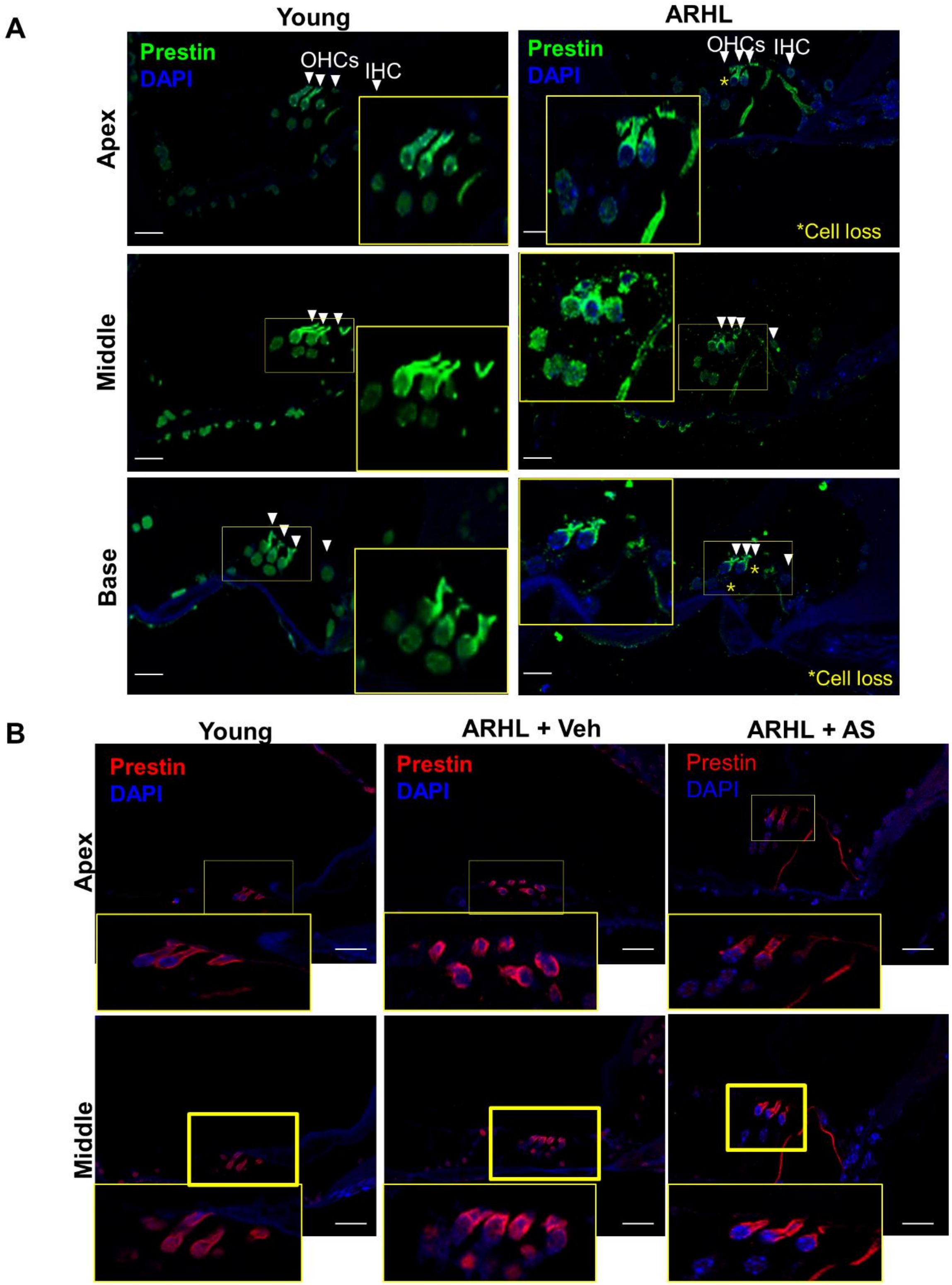

Figure S6. AS prevents alteration of OHC formation and prestin membrane aggregation in ARHL cochlea. (**A and B**) Cochlear sections from young and ARHL mice, treated or not with AS (1.2 mg/kg), were immunolabeled with anti-prestin antibody to evaluate prestin

rearrangement in the organ of Corti (yellow box). DAPI was used as the counterstain. Scale bars, 20 μm. White arrowhead: OHC and IHC. *: Loss of cell.

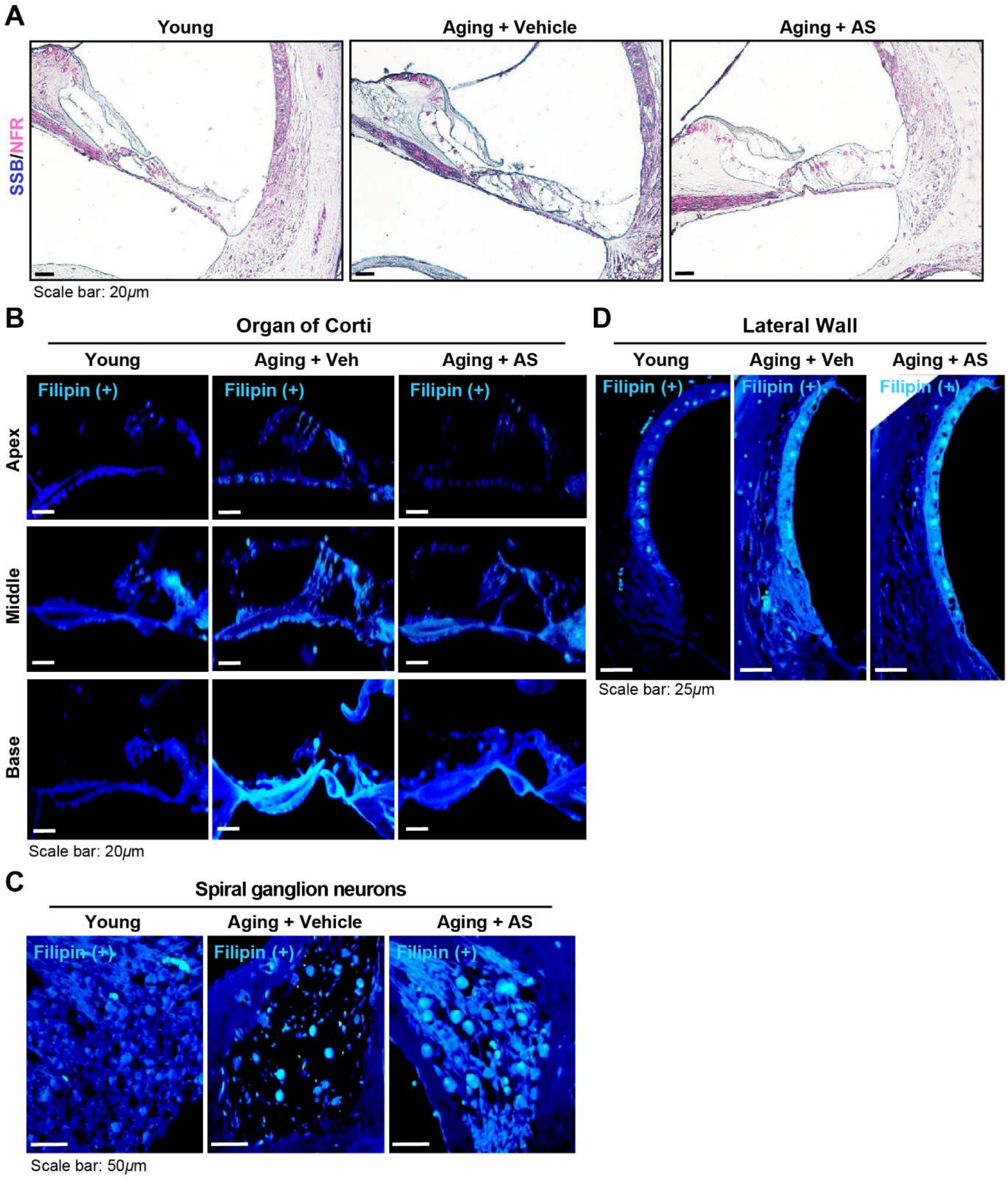

Figure S7. AS decreases lipofuscin and cholesterol levels in ARHL, as compared to young mice. (**A**) Sudan Black B (SBB) staining with Nuclear Fast Red (NFR) counterstaining in cochlear sections from young and ARHL mice treated or not with AS. (**B**) Filipin staining from the apex to the basal turn of the organ of Corti, (**C**) spiral ganglion neurons, and (**D**) lateral walls in young and aging mice.

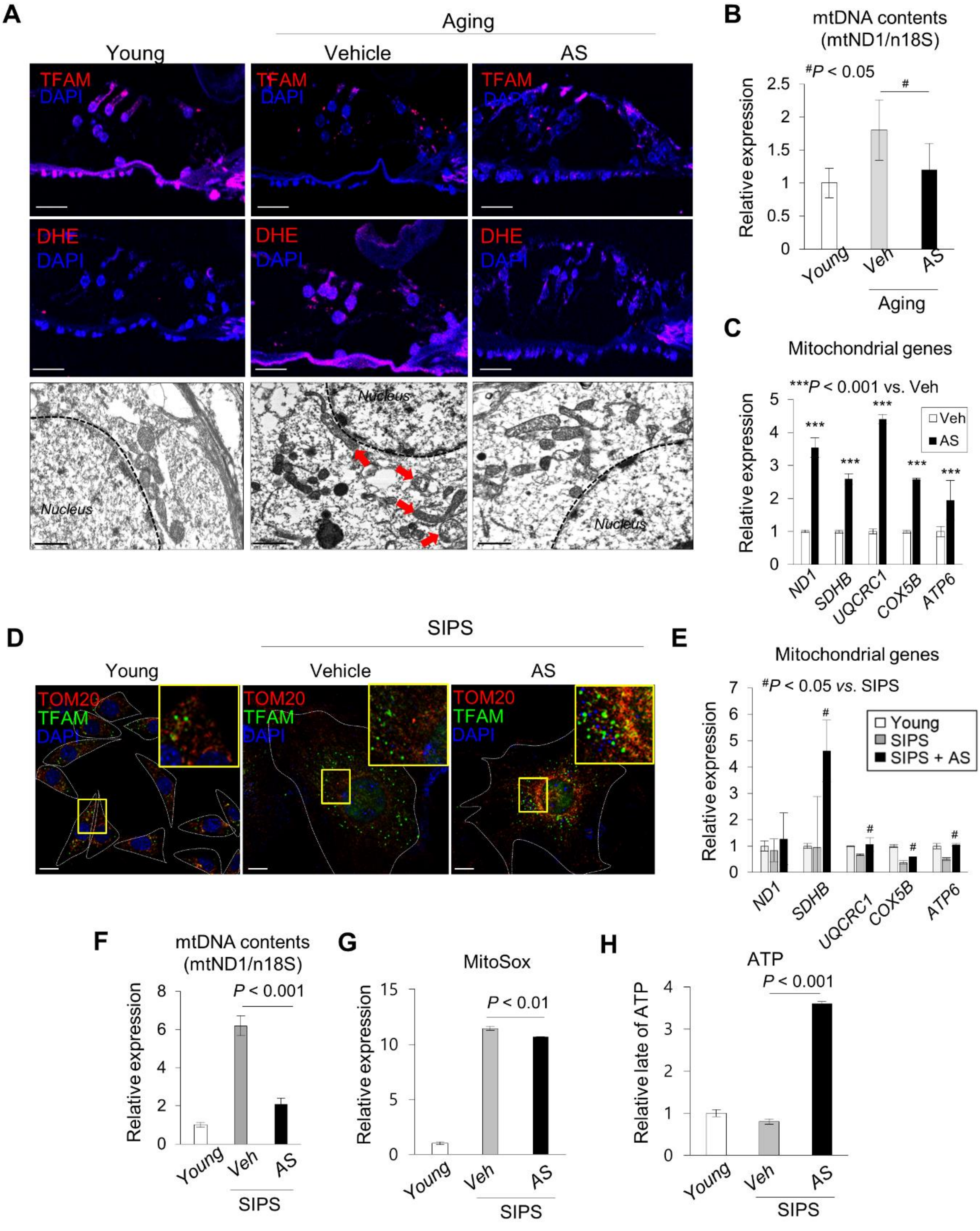

Figure S8. Atorvastatin (AS) balancing intracellular reactive oxygen species (ROS) and adenosine triphosphate (ATP) production by regulation of antioxidants and mitochondria biogenesis depend on TFEB expression. (**A**) Cochlear sections from vehicle- or AS-treated young and ARHL mice were immunolabeled with an anti-TFAM antibody to evaluate

mitochondrial biogenesis. The fluorescent probe, dihydroethidium (DHE; 10 μM), was used to detect ROS. DAPI was used as the counterstain. Scale bars, 20 μm. Abnormal mitochondria and lipofuscin aggregates observed by transmission electron micrograph (TEM) in the cochlea of vehicle or AS-treated young and ARHL mice. Red arrow indicates cristae loss and alteration of mitochondria shape. Scale bar, 1 μm. (**B**) Total DNA was isolated from whole cochlea extract, and mtDNA was quantified by qPCR as the ratio of mtDNA (NADH-ubiquinone oxidoreductase subunit 1; ND1) to nDNA. (**C**) qPCR of mitochondrial OXOPHOS complex genes. 18S ribosomal RNA was used as the control. Means ± SD. (**D**) HEI-OC1 cells were treated with DOXO (100 ng/mL) for 24 h and treated with vehicle or AS (0.5 μM) for 3 days. TOM20 (ab56783) and TFAM protein levels were evaluated by immunocytochemistry. Scale bars, 20 μm. DAPI was used as the counterstain. White dotted lines show cell bodies. Yellow boxes show mitochondrial biogenesis. (**E**) qPCR of mitochondrial OXOPHOS complex genes. 18S ribosomal RNA was used as the control. Means ± SD. (**F**) mtDNA content quantified by qPCR. (**G**) Dysfunctional mitochondrial ROS generation detected by MitoSOX (5 μM) staining and flow cytometry. (**H**) Total ATP content was measured using an ATP Determination Kit. Experiments were performed at least three times for each condition and repeated at least twice. $p < 0.05$, $p < 0.01$, $p < 0.001$ (Student's *t*-test or one-way ANOVA followed by Tukey's HSD test).

Supplemental References

1. Carey KL, Paulus GLC, Wang L, et al. TFEB transcriptional responses reveal negative feedback by BHLHE40 and BHLHE41. Cell Rep. 2020 Nov 10;33(6):108371. PubMed PMID: 33176151.
2. Martina JA, Diab HI, Lishu L, et al. The nutrient-responsive transcription factor TFE3 promotes autophagy, lysosomal biogenesis, and clearance of cellular debris. Sci Signal. 2014 Jan 21;7(309):ra9. PubMed PMID: 24448649.
3. Wang Y, Huang Y, Liu J, et al. Acetyltransferase GCN5 regulates autophagy and lysosome biogenesis by targeting TFEB. EMBO Rep. 2020 Jan 7;21(1):e48335. PubMed PMID: 31750630.
